# Identification of Bis-Cyclic Guanidines as Antiplasmodial Compounds from Positional Scanning Mixture-Based Libraries

**DOI:** 10.3390/molecules24061100

**Published:** 2019-03-20

**Authors:** David L. Perry, Bracken F. Roberts, Ginamarie Debevec, Heather A. Michaels, Debopam Chakrabarti, Adel Nefzi

**Affiliations:** 1 Division of Molecular Biology and Microbiology, Burnett School of Biomedical Sciences, University of Central Florida, Orlando, FL 32826, USA; dperryII@Knights.ucf.edu (D.L.P.J.); bracken.roberts@knights.ucf.edu (B.F.R.); dchak@ucf.edu (D.C.); 2 Torrey Pines Institute for Molecular Studies, 11350 SW Village Parkway, Port Saint Lucie, FL 34987, USA; gdebevec@tpims.org; 3 Smart Biomolecules, Inc. 11350 SW Village Parkway, Port Saint Lucie, FL 34987, USA; hmichaels@tpims.org

**Keywords:** heterocyclic peptidomimetics, parallel synthesis, combinatorial chemistry, guanidines, solid-phase synthesis, antiplasmodial, malaria

## Abstract

The screening of more than 30 million compounds derived from 81 small molecule libraries built on 81 distinct scaffolds identified pyrrolidine bis-cyclic guanidine library (TPI-1955) to be one of the most active and selective antiplasmodial libraries. The screening of the positional scanning library TPI-1955 arranged on four sets of sublibraries (26 + 26 + 26 + 40), totaling 120 samples for testing provided information about the most important groups of each variable position in the TPI-1955 library containing 738,192 unique compounds. The parallel synthesis of the individual compounds derived from the deconvolution of the positional scanning library led to the identification of active selective antiplasmodial pyrrolidine bis-cyclic guanidines.

## 1. Introduction

Malaria globally still causes 216 million clinical cases and ~445,000 deaths each year, but the drugs that are available for treatment are rapidly losing their efficacy because of widespread prevalence of drug resistant parasites [1,2]. Malaria contributes significantly to overall childhood mortality in the poorest nations. The global economic toll of malaria is enormous, with direct costs estimated at $12B per year in addition to an estimated 1.3% reduction in economic growth in countries that bear a heavy malaria burden. The appearance of parasites resistant to artemisinin derivatives in wide areas of Southeast Asia underscores the fragility of the available malaria treatment measures. Given the global toll of malaria, it is urgent to identify novel leads directed against new cellular targets for the next generation of malaria therapeutics [3,4]. Although there has been recent progress in malaria therapeutics development, the situation still remains fragile because of the ability of the parasite to rapidly develop resistance [5,6,7,8]. Therefore, to find a solution to the problem of resistance, even to future malaria medicines, there is an urgent need to continue with the development of novel leads to create a pipeline of malaria therapeutics, directed against new cellular targets [9]. Historically, natural products and their derivatives have had major impacts in drug discovery, particularly for infectious diseases [10,11]. Artemisinin and its semisynthetic derivatives are prime examples in the malaria arena. However, such chemotypes remain severely underrepresented in modern screening campaigns due to the synthetic challenges associated with these complex stereochemically-rich molecules. We screened Torrey Pines Institute for Molecular Studies (TPIMS) proprietary high-density combinatorial libraries toward the discovery of novel antimalarial lead compounds [12,13]. The TPIMS collection of libraries contains a large number of small molecule compounds residing largely in the underexplored areas of chemical space [12,13,14,15,16].

## 2. Results and Discussion

Each of the 81 small molecule libraries is arranged in positional scanning format [17]. Each of the libraries is made up of 100–200 mixture samples. These mixture samples are systematically formatted so that activities of individual compounds can be predicted from the screening of exponentially fewer samples.

Our screening of 30 million compounds from 81 libraries built on 81 distinct scaffolds identified bis-cyclic guanidine compounds (TPI-1955) to be one of the most active and selective antiplasmodial compounds. To assess the antiplasmodial potency of the TPIMS small molecule compounds, we pursued an unbiased cell-based screening of TPIMS Scaffold Ranking Library of 30 million compounds using the SYBR green I-based DNA quantification assay inhibiting malaria parasite proliferation [18,19,20]. The scaffold ranking library of 30 million compounds provides a method to triage the available TPIMS libraries based on activity in a given assay [13,21,22]. The scaffold ranking library is composed of 81 samples forming different small molecule positional scanning libraries. Each sample of the 81 pharmacophores contains an approximate equal molar amount of each compound in that library. As an example, the sample of the selected bis-imidazolidinimine library 1955 is composed of 738,192 compounds in equimolar amounts. These samples (all mixtures) were directly prepared in parallel to the library synthesis or can be prepared by mixing the 120 samples forming the complete positional scanning library [21]. Use of scaffold ranking library approach was successfully used in many assays including, the identification of metalloprotease ADAM 17 ligands [23], the identification of novel neurite outgrowth promoters with nanomolar potency [24], the discovery of small molecule inhibitors of human As(III) S-adenosylmethionine methyltransferase (AS3MT) [25] and the identification of new analgesics through the direct in vivo screening assay [26].

**Figure 1:**
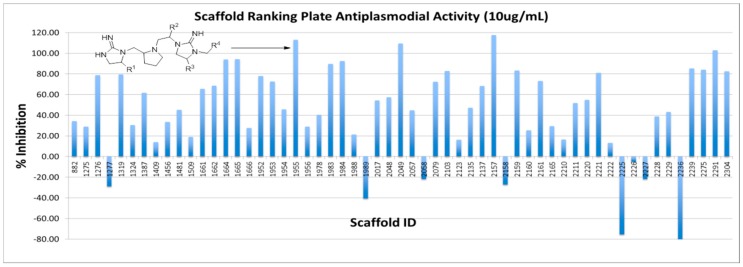
Screening of scaffold ranking library for antiplasmodial activity.

As our main goal was to overcome the problem of drug-resistant malaria, we used the chloroquine (CHQ)-resistant Pf Dd2 strain. The assays were done in 96-well plates under standard culture conditions for 72 h as described in the experimental section [18,19,20]. All plates contained control wells: (a) 0.1% dimethyl formamide (DMF) solvent control and (b) 1 µM and 10 nM chloroquine. Z-factors were calculated, and only those assays with values >0.7 were considered for evaluation. We also monitored for any autofluorescence and cell lysis properties of the compounds. The library mixtures contained between 2016 individual compounds and 18.9 million individual compounds. Compounds in the mixtures are present in equimolar concentrations. Figure 1 shows results of the screening of TPIMS scaffold ranking libraries at 10 µg/mL. Fourteen libraries exhibited >80% inhibition of Pf Dd2 growth upon 72 h exposure, of which four libraries (2275, 1955, 2291, and 2157) showed good selectivity (>15 fold) when tested for cytotoxicity against HepG2 human hepatoma cells. Based on the results of the scaffold ranking library plate and structural features, we selected the pyrrolidine bis-cyclic guanidine library TPI-1955 positional scanning library for subsequent deconvolution [17,27].

**Figure 2:**
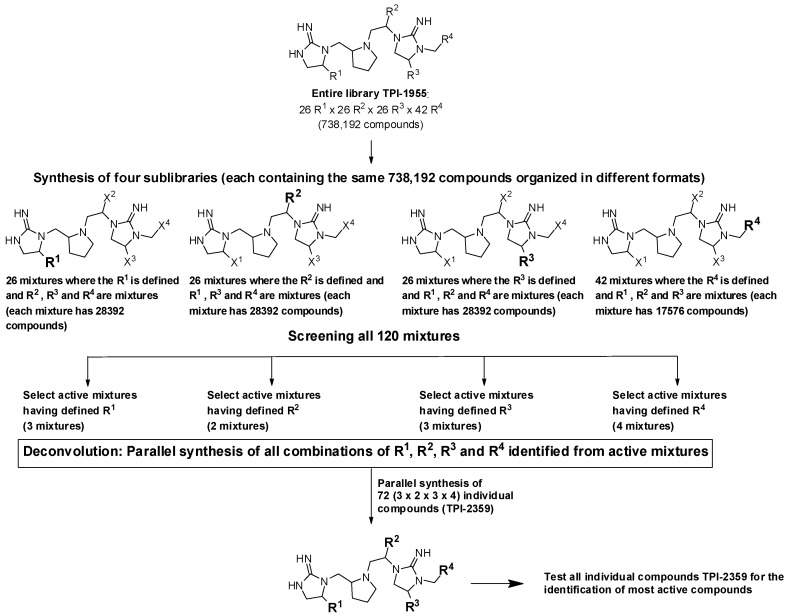
Deconvolution of positional scanning pyrrolidine bis-cyclic guanidine library TPI-1955 and synthesis of individual compounds TPI-2359.

We have screened 120 mixture samples of the 1955 positional scanning library (bis-cyclic guanindine) with a total diversity of 738,192 compounds. The mixture samples from positional scanning libraries were evaluated at 500 ng/mL per mixture. The selection of the most active mixtures guides the synthesis of individual compounds. As outlined in Figure 2, a Positional Scanning Synthetic Combinatorial Library (TPI-1955), having four sites of diversity, consisting of four separate sub-libraries, each having a single defined position (R), and mixture positions (X) [17,27]. The figure illustrates the use of such a library to identify the most active groups at each position of the selected pyrrolidine bis-cyclic guanidine library (TPI-1955) directly from the initial screening data.

**Scheme 1:**
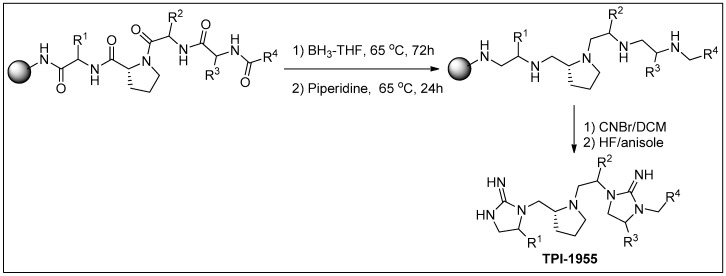
Synthesis of pyrrolidine bis-cyclic guanidines from resin-bound acylated tetrapeptides.

**Figure 3:**
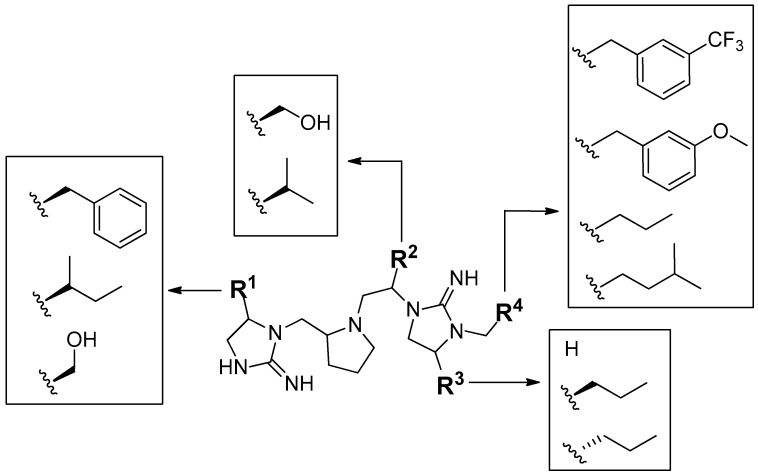
Identification of active groups at positions R^1^–R^4^ in the compound mixture.

The screening of the four sets of mixtures, totaling 120 samples for testing (26 + 26 + 26 + 40), provided information about the most important groups of each variable position in the TPI-1955 library containing 738,192 unique compounds. Three mixtures with defined position R^1^ corresponding to the amino acids (l-phenylalanine, l-isoleucine and l-serine), two mixtures with defined position R^2^ corresponding to the amino acids (l-serine and l-valine), three mixtures with defined position R^3^ corresponding to the amino acids (glycine, l-norvaline, and d-norvaline), and four mixtures with defined position R^4^ corresponding to the carboxylic acids (meta trifluoromethyl tolyl acetic acid, 3-methoxyphenylacetic acid, butyric acid, and 4-methylvaleric acid) yielded good inhibition activity. The parallel synthesis of all individual compounds named TPI-2359 derived from the deconvolution of TPI-1955 representing all the combination (3 × 2 × 3 × 4 = 72) of active mixtures with defined R^1^, R^2^, R^3^, and R^4^ (Figure 3) was performed using the strategy outlined in Scheme 1. The data shows significant differentiation in activity levels among the samples tested at each position, a key feature for deconvoluting a positional scanning library. Moreover, some preliminary SAR can be seen from the library screening. High activity is observed with compounds having small aliphatic groups at positions R^1^, R^2^, and R^3^, and aromatic and aliphatic groups in position R^4^. The (S) sec-butyl group is preferred in position R^1^, while the (S) isopropyl group and (S) hydroxymethyl are preferred in position R^2^. In position R^3^ the n-propyl is preferred with no preference of configuration. Aromatic and linear aliphatic groups are both preferred in position R^4^.

**Figure 4:**
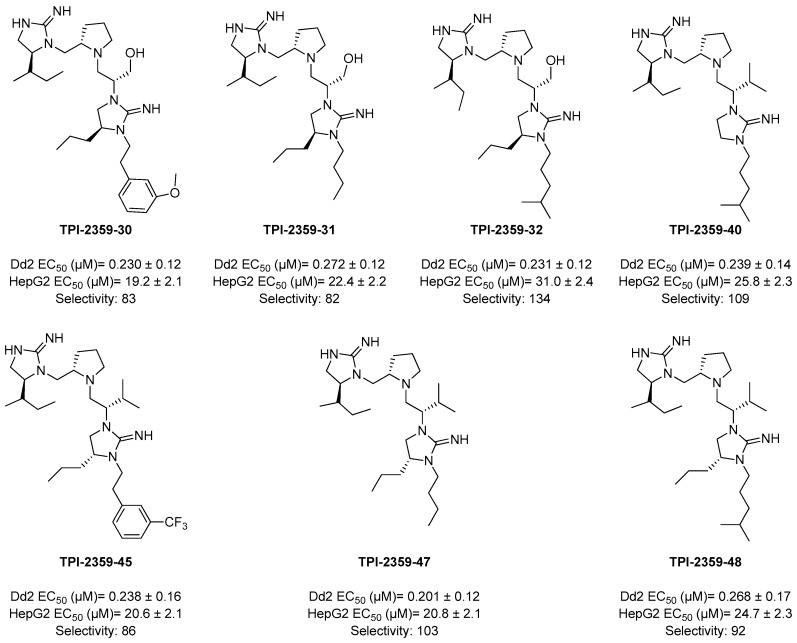
Structures and activity of identified top antiplasmodial hits.

The identified bis-cyclic guanidine hits TPI-2359 derived from the deconvolution of the library TPI-1955 were prepared in parallel starting from resin-bound amino acids (diversity R^1^) [28]. Boc-proline was coupled using standard solid-phase peptide synthesis (SPPS) coupling reagents, followed by Boc deprotection and subsequent coupling of two Boc-amino acids (diversities R^2^ and R^3^). The N-terminal Boc was cleaved and the generated primary amine was N-acylated with different commercially available carboxylic acids (diversity R^4^). The generated resin-bound N-acylated tetrapeptide was exhaustively reduced using borane-THF. Our approach involved the use of proline as a spacer, which, following the exhaustive reduction of the amide groups, yielded a resin-bound pentaamine containing two pairs of secondary amines separated by a pyrrolidine ring. The resulting pairs of secondary amines were treated with cyanogen bromide to generate, following intramolecular cyclization, the corresponding resin-bound pyrrolidine- bis-cyclic guanidines. All the compounds were purified and screened against the Pf Dd2 strain, compounds with EC_50_ activity ranging between 201 and 350 nM were identified (Figure 4). As a counterscreen, we evaluated the cytotoxicity of these compounds in human hepatocyte cell line HepG2 using MTS cell proliferation assay [29]. The compounds exhibited very promising selectivity of >60. 

**Figure 5:**
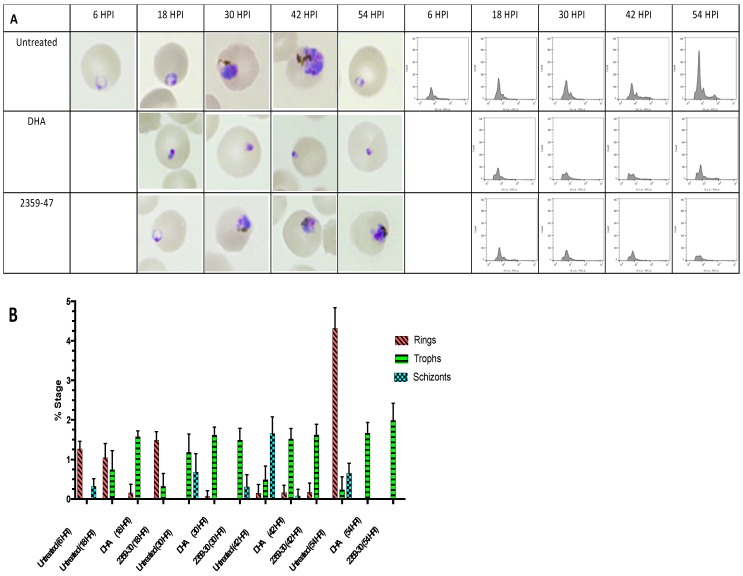
Stage-specific inhibition of Plasmodium growth by TPI 2359-47. Tightly synchronized parasites were treated at 6-h post-invasion of merozoites with 5 × EC_50_ concentration of the chemotype. (**A**) Microscopic evaluation of Giemsa-stained thin smears in the left. Histogram plot of YOYO-1 labeled cells following treatment of the compound were shown in the right. (**B**) Distribution of developmental stages at different time intervals following treatment of the compound at 6 h postinvasion. Three replicates of 1000 parasitized erythrocytes were counted to determine stage-specific distribution. DHA, dihydroartemisinin.

**Figure 6:**
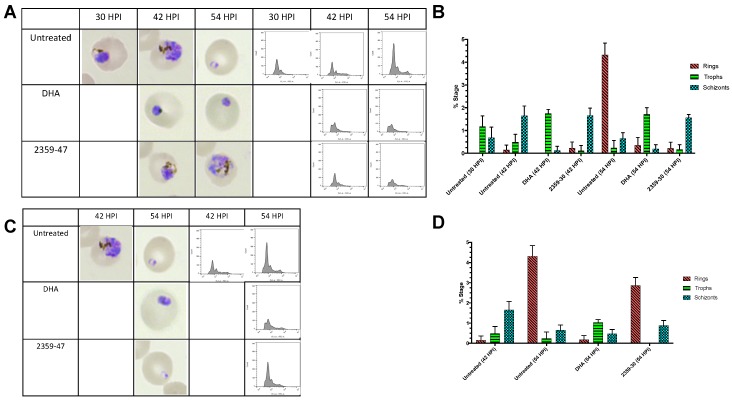
Stage-specific inhibition of Plasmodium growth by TPI 2359-47. (**A**) Microscopic evaluation of Giemsa-stained thin smears and flow cytometric analysis of the YOYO-1-labeled cells to measure DNA content when the culture was exposed to the inhibitor (5 × EC_50_) at 30 h postinvasion. (**B**) Distribution of different stages when the culture was treated at 30 h postinvasion. (**C**,**D**) Effect following exposure of the parasite to the compound at 42 h postinvasion. Three replicates of 1000 parasitized erythrocytes were counted to determine stage-specific distribution. DHA, dihydroartemisinin.

To gain insight into the mechanism of action of this scaffold in Plasmodium, we determined the stage-specificities of one of the best analogs—TPI 2359-47—by testing its effects on intraerythrocytic development of the parasite. A tightly synchronized Pf Dd2 culture was exposed to 5 × EC_50_ concentration of the compound at 6- (ring stage), 18- (trophozoite stage), 30- (schizont stage), and 42-h following invasion. The effect of TPI 2359-47 on developmental stage progression was assessed by flow cytometry of fixed YOYO-1 stained Pf cells collected at designated time intervals. As seen in Figure 5A, the control culture matured, as expected, to rings 54-h postinvasion. In contrast, the maturation of the TPI 2359-47 treated culture was blocked. This was clearly evident from the flow cytometric data presented in the right panel. Figure 5B shows that the inhibitor cells did not mature beyond the trophozoite stages. Treatment of the culture at 18 h postinvasion had similar effects (data not shown). Interestingly, exposing the culture to TPI 2359-47 at 30 h postinvasion blocks at the schizont stage (Figure 6A,B), while the treatment at the segmenter stage at 42 h had no effect on maturation (Figure 6C,D). This suggests that TPI 2359-47 acts on a cellular target whose activity is important for parasite maturation during ring through schizont stages but not at the segmenter stage. In addition, we checked the plasma stability of the compound TPI 2359-47. The compound was incubated in rat plasma for up to 5 h and no significant degradation was detected. Different bis-cyclic guanidine libraries, including TPI-1955, were screened in different assays including inhibitors of tyrosine recombinases and Holliday junction-resolving enzymes [30], leishmaniasis [31], and bacteria [32,33], and their deconvolution led to the identification of very active compounds in each assay. The activities of the individual compounds we have found at each target are specific to the given molecules identified. The individual compounds identified following the positional scanning deconvolution of the library are unique for each assay. Typically, none of the synthesized individual compounds for a specific target had significant activity at other targets. Thus, the activity is a combination of the scaffold itself and the differing substituent R groups. For example, a simple change of the stereochemistry (l and d amino acids) usually leads to loss of activity. As is the same as for the reported thousands of active peptides against infectious diseases, including malaria, antimicrobials, and leishmaniasis, the vast majority of them all contain a significant combination of positively charged and aromatic amino acids, such as lysine and/or arginine as well as tryptophan and/or phenylalanine. The positively charged amino acid residues, such as arginine and lysine, were shown to play an important role in the activity of antimicrobial peptides as a result of the electrostatic interactions between the identified peptides and bacterial membranes [34,35,36]. Of importance, not all peptides which contain these amino acids have significant activity. It is the combination of the location of the functional groups with the location on the central scaffold and the peptide conformation that drive the specific activity. The same is true for the various nonpeptide bis-cyclic guanidines we have synthesized and tested. It is not surprising to find active compounds from such libraries, but the majority of the compounds have little to no activity. Thus, we have thousands of related analogs that are not active.

## 3. Experimental

All the reagents, amino acids, and solvents were commercial grade. LC-MS (ESI) traces were recorded on samples with concentrations of 1 mg/ mL in 50:50 MeCN/ water at both 214 nm and 254 nm using a reverse phase Vydac column with a gradient of 5 to 95% formic acid in MeCN. The purity of the crude samples was estimated based on the UV traces recorded. Hydrofluoric acid cleaves were performed in specially equipped and ventilated hoods with full personal protective equipment. All synthesized compounds were purified by RP-HPLC (Shimadzu Prominence HPLC system, Kyoto, Japan). The purity of all final compounds was >95%.

Chemistry General: ^1^H-NMR spectra were recorded in DMSO-*d*_6_ relative to internal reference solvent peak for 1H-NMR (500 MHz) and ^13^C-NMR (125 MHz). NMR chemical shifts are expressed in ppm relative to internal solvent peak and coupling constants were calculated in hertz. The reported final purity of the compounds were verified by Shimatzu HPLC and mass spectra under the following conditions; column, Phenomenex Luna 150 × 21.20 mm, 5 micron, C18; mobile phase, (A) H_2_O (+0.1% Formic acid)/(B) MeCN (+0.1% Formic acid), and 3 gradient methods used based on compound hydrophobicity (2% B to 20% B, 11 min; 25% B to 45% B, 31 min; 45% B to 65% B, 21 min); flow rate, 12 mL/min; detection, UV 214 nm. The purity of all final compounds was >95%.

All chirality data was generated from the corresponding amino acids. Under our reaction conditions the epimerization is minimized to less than 5% [37,38,39].

General synthesis of the pyrrolidine bis-cyclic guanidine compounds: All compounds were synthesized following the strategy outlined in Scheme 1. The solid-phase synthesis was performed using the “tea-bag” methodology [28].

Parallel synthesis of resin-bound acylated tetrapeptides (**1**): Initially, 100 mg of *p*-methylbenzdrylamine (*p*MBHA) resin per compound (1.1 mmol/g, 100–200 mesh) was sealed in a mesh “tea-bag,” neutralized with 5% diisopropylethylamine (DIEA) in dichloromethane (DCM), and subsequently swelled with additional DCM washes. The first diversity position (R^1^) was introduced by the coupling of Boc amino acid (6 eq) in Dimethylformamide (0.1 M DMF) for 60 min in the presence of Diisopropylcarbodiimide (DIC, 6 equiv.) and 1-Hydroxybenzotriazole hydrate (HOBt, 6 equiv.). The Boc protecting group was removed with 55% TFA/DCM for 30 min and subsequently neutralized with 5% DIEA/DCM (3×). The second amino acid proline and third and fourth amino acid diversity positions (R^2^ and R^3^) were introduced by the subsequent couplings of Boc-amino acids utilizing the same standard coupling procedures. The N-terminal Boc protecting group was removed with 55% Trifluoroacetic Acid (TFA) in DCM for 30 min and, subsequently, neutralized with 5% DIEA/DCM (3×). Substitutions to the fourth diversity position (R^4^) were introduced following the coupling of a carboxylic acid, diversity R^4^ (10 eq) in the presence of DIC (10 eq). All coupling reactions were monitored for completion by Ninhydrin test.

Synthesis of chiral tetraamines: The reduction was performed on solid-phase in a 1000 mL Wilmad LabGlass vessel under nitrogen in the presence of 1.0 M Borane-Tetrahydrofuran (BH_3_-THF) complex solution. A 40-fold excess of BH_3_-THF was used for each amide bond. The vessel containing the 16 bags was heated to 65 °C and the temperature was maintained for 72 h [40,41]. The solution was then discarded and the bags were washed with THF and methanol. Once completely dry, the bags were treated overnight with piperidine at 65 °C and washed several times with methanol, DMF, and DCM. Before proceeding, completion of the reduction was monitored by a control cleavage and analyzed by LCMS interface (Applied Biosystems/MDS Sciex, Darmstadt, Germany; and a Shimadzu Prominence HPLC system. Kyoto, Japan).

Synthesis of the desired pyrrolidine bis-cyclic guanidines: Our approach involved the use of proline as a spacer, which, following the exhaustive reduction of the amide groups, yielded resin-bound pentaamine containing two pairs of secondary amines separated by a pyrrolidine ring. The resin-bound pentaamines were treated with 10 eq of cyanogen bromide (CNBr) in anhydrous DCM. The cleavage of the compounds from the resin packets is performed with hydrogen fluoride (HF) (approximately 5 mL of HF per resin packet containing up to 0.225 mmol of resin-bound compound with 0.35 mL anisole added as a scavenger; 90 min, 0 °C) by using a 24 vessel HF cleavage apparatus. After HF evaporation, the resulting individual compounds were extracted by sonicating with 50% aqueous acetonitrile (3 × 5 mL), frozen and lyophilized. The crude compounds were then dissolved in acetic acid and lyophilized (two times). The obtained white powders were purified using preparative high performance liquid chromatography and the desired compounds were obtained in good yield and high purity.

*(R)-3-((S)-2-(((S)-5-((S)-sec-butyl)-2-iminoimidazolidin-1-yl)methyl)pyrrolidin-1-yl)-2-((S)-2-imino-3-(3-methoxyphenethyl)-4-propylimidazolidin-1-yl)propan-1-ol* (**TPI-2359-30**): ^1^H-NMR (400 MHz, DMSO-*d*_6_, 300 K) 8.04 (bs, 1H), 7.22–7.28 (m, 2H), 6.86–7.40 (m, 1H), 6.82 (dd, *J* = 8.1 Hz, *J* = 1.5 Hz, 1H), 4.09–4.14 (m, 1H), 3.70–3.80 (m, 2H), 3.44–3.60 (m, 2H), 3.20–3.38 (m, 2H), 2.72–2.89 (m, 6H), 2.55 (t, *J* = 1.7 Hz, 1H), 2.46 (t, *J* = 1.7 Hz, 1H), 2.25–2.38 (m, 4H), 1.82–1,92 (m, 2H), 1.60–1.78 (m, 4H), 1.36–1.50 (m, 4H), 1.20–1.35 (m, 4H), 1.13–1.18 (m, 1H), 1.04 (d, *J* = 6.0 Hz, 1H), 0.92 (dd, *J* =16.0 Hz, *J* = 7.3 Hz, 3H), 0.7 (d, *J* = 8.3 Hz, 3H). ^13^C-NMR (100 MHz, DMSOD_6_, 300 K) 174.9, 167.1, 160.0, 159.8, 157.3, 156.3, 140.1, 129.7, 121.6, 114.9, 112.5, 62.8, 62.6, 59.5, 57.8, 56.3, 55.9, 55.4, 53.8, 48.9, 56.4, 45.7, 44.7, 43.5, 41.0, 34.1, 33.7, 33.2, 28.2, 25.1, 23.4, 17.4, 14.2, 12.2, 11.5. MS (ESI) *m*/*z* [M + H]^+^: 542.0.

*(R)-3-((S)-2-(((S)-5-((S)-sec-butyl)-2-iminoimidazolidin-1-yl)methyl)pyrrolidin-1-yl)-2-((S)-3-butyl-2-imino-4-propylimidazolidin-1-yl)propan-1-ol* (**TPI-2359-31**): ^1^H-NMR (400 MHz, DMSO-*d*_6_, 300 K) 8.43 (s, 1H), 4.07–4.65 (m, 1H), 3.88–3.94 (m, 1H), 3.61–3.76 (m, 2H), 3.43–3.56 (m, 2H), 3.36–3.38 (m, 2H), 3.31–3.35 (m, 1H), 3.10–3.16 (m, 1H), 3.05–3.09 (m, 2H), 2.89–2.94 (m, 1H), 2.71–2.79 (m, 1H), 2.30–2.39 (m, 1H), 2.28–2.30 (m, 1H), 1.81–1.87 (m, 2H), 1.61–1.68 (m, 4H), 1.40–1.59 (m, 6H), 1.22–1.44 (m,6H), 1.09–1.16 (m, 1H), 0.90–0.99 (m, 9H), 0.73 (d, *J* = 6.4 Hz, 3H). ^13^C-NMR (100 MHz, DMSOD_6_, 300 K) 167.3, 159.9, 157,4, 62.8, 62.6, 59.4, 56.1, 55.3, 53.8, 46.2, 44.7, 42.1, 40.6, 34.1, 33.6, 29.3, 28.2, 25.1, 23.3, 19.6, 17.4, 15.3, 14.2, 12.2, 11.5. MS (ESI) *m*/*z* [M + H]^+^: 463.0.

*(R)-3-((S)-2-(((S)-5-((S)-sec-butyl)-2-iminoimidazolidin-1-yl)methyl)pyrrolidin-1-yl)-2-((S)-2-imino-3-(4-methylpentyl)-4-propylimidazolidin-1-yl)propan-1-ol* (**TPI-2359-32**): ^1^H-NMR (400 MHz, DMSO-*d*_6_, 300 K) 8.42 (s, 1H), 4.03–4.07 (m, 1H), 3.83–97 (m, 1H), 3.60–3.69 (m, 1H), 3.28–3.38 (m, 3H), 3.22–3.26 (m, 1H), 3.07–3.18 (m, 3H), 2.81–2.98 (m, 2H), 2.68–2.72 (m, 2H), 2.22–2.38 (m, 3H), 1.85–1.89 (m, 2H), 1.66–1.71 (m, 2H), 1.51–1.55 (m, 3H), 1.39–1.46 (m, 4H), 1.22–1.26 (m, 4H), 1.09–1.60 (m, 5H), 0.91 (t, *J* = 6.8 Hz, 3H), 0.86 (d, *J* = 6.6 Hz, 6H), 0.72 (d, *J* = 4.6 Hz, 3H). ^13^C-NMR (100 MHz, DMSOD6, 300 K) 167.3, 159.9, 157.3, 156.4, 62.8, 62.5, 59.4, 56.1, 56.1, 55.3, 53.8, 49.0, 46.2, 44.7, 42.5, 41.0, 35.4, 34.1, 33.7, 28.2, 27.7, 25.0, 24.0, 23.4, 17.3, 15.3, 14.2, 12.2, 11.5. MS (ESI) *m*/*z* [M + H]^+^: 492.0.

*1-((S)-1-((S)-2-(((S)-5-((S)-sec-butyl)-2-iminoimidazolidin-1-yl)methyl)pyrrolidin-1-yl)-3-methylbutan-2-yl)-3-(4-methylpentyl)imidazolidin-2-imine* (**TPI-2359-40**): ^1^H-NMR (400 MHz, DMSO-*d*_6_, 300 K) 8.44 (s, 1H), 4.03–4.35 (m, 2H), 3.59–3.68 (m, 2H), 3.28–3.38 (m, 2H), 3.04–3.08 (m, 2H), 2.88–2.96 (m, 2H), 2.55–2.62 (m, 1H), 2.42–2.49 (m, 1H), 1.00–1.90 (m, 2H), 1.68–1.78 (m, 2H), 1.42–1.58 (m, 6H), 1.20–1.36 (m, 1H), 1.10–1.19 (m, 4H), 0.97 (d, *J* =6.4 Hz, 3H), 0.91 (t, *J* = 7.3 Hz, 3H), 0.87 (d, *J* = 3.6 Hz, 6H), 0.82–0.87 (m, 3H), 0.75 (d, *J* = 6.8 Hz, 3H). ^13^C-NMR (100 MHz, DMSOD6, 300 K) 174.4, 167.0, 160.3, 157.6, 62.9, 62.7, 59.5, 54.4, 54.0, 46.1, 45.2, 44.8, 41.3, 41.1, 35.3, 34.3, 29.4, 28.2, 27.6, 25.2, 24.4, 23.7, 23.5, 22.9, 22.8, 19.8, 19.4. MS (ESI) *m*/*z* [M + H]^+^: 462.0.

*(R)-1-((S)-1-((S)-2-(((S)-5-((S)-sec-butyl)-2-iminoimidazolidin-1-yl)methyl)pyrrolidin-1-yl)-3-methylbutan-2-yl)-4-propyl-3-(3-(trifluoromethyl)phenethyl)imidazolidin-2-imine* (**TPI-2359-45**): ^1^H-NMR (400 MHz, DMSO-*d*_6_, 300 K) 8.44 (s, 1H), 7.75 (s, 1H), 7.67 (d, *J* = 7.3 Hz, 1H), 7.61 (d, *J* = 7.6 Hz, 1H), 7.50–7.60 (m, 1H), 4.02–4.08 (m, 1H), 3.78–3.91 (m, 1H), 3.65–3.68 (m, 2H), 3.43–3.58 (m, 3H), 3.29–3.41 (m, 2H), 3.10–3.19 (m, 2H), 2.80–3.19 (m, 4H), 2.56–2.60 (m, 1H), 2.36–2.39 (m, 2H), 1.78–1.92 (m, 4H), 1.64–1.68 (m, 2H), 1.42–1.51 (m, 2H), 1.22–1.38 (m, 4H), 1.08–1.2 (m, 2H), 0.97 (d, *J* = 6.5 Hz, 3H), 0,91 (dd, *J* = 13.5 Hz, *J* = 6.7 Hz, 6H), 0.83 (d, *J* = 6.6 Hz, 3H), 0.749 (d, *J* = 6.6 Hz, 3H). ^13^C-NMR (100 MHz, DMSOD6, 300 K) 167.3, 160.2, 157.2, 139.9, 133.7, 129.7, 126.1, 123.6, 63.1, 62.7, 59.4, 56.2, 54.4, 54.1, 46.7, 46.3, 43.1, 41.2, 34.4, 33.8, 32.4, 29.5, 28.3, 25.1, 23.9, 23.7, 19.8, 19.4, 17.6, 14.2, 12.1, 11.7. MS (ESI) *m*/*z* [M + H]^+^: 592.0.

*(R)-3-butyl-1-((S)-1-((S)-2-(((S)-5-((S)-sec-butyl)-2-iminoimidazolidin-1-yl)methyl)pyrrolidin-1-yl)-3-methylbutan-2-yl)-4-propylimidazolidin-2-imine* (**TPI-2359-47**): ^1^H-NMR (400 MHz, DMSO-*d*_6_, 300 K) 8.20 (s, 1H), 4.02–4.08 (m, 2H), 3.90–3.97 (m, 1H), 3.71–3.89 (m, 1H), 3.25–3.45 (m, 4H), 3.15–3.19 (m, 2H), 2.01–2.03 (m, 2H), 1.68–1.86 (m, 10H), 1.48–1.56 (m, 2H), 1.30–1.41 (m, 2H), 1.20–1.30 (m, 4H), 1.15–1.20 (m, 2H), 0.91–0.99 (m, 6H), 0.90 (d, *J* = 6.9 Hz, 6H), 0.82 (d, *J* = 6.6 Hz, 3H), 0.73 (d, *J* = 6.6 Hz, 3H). ^13^C-NMR (100 MHz, DMSOD6, 300 K) 167.0, 160.3, 157.4, 63.1, 62.6, 59.3, 55.8, 54.1, 46.5, 46.1, 41.9, 41.2, 34.4, 33.9, 29.4, 28.8, 28.4, 25.2, 23.7, 23.6, 19.9, 19.8, 19.5, 17.5, 14.2, 14.1, 12.1, 11.8. MS (ESI) *m*/*z* [M + H]^+^: 476.0

*(R)-1-((S)-1-((S)-2-(((S)-5-((S)-sec-butyl)-2-iminoimidazolidin-1-yl)methyl)pyrrolidin-1-yl)butan-2-yl)-3-(4-methylpentyl)-4-propylimidazolidin-2-imine* (**TPI-2359-48**): ^1^H-NMR (400 MHz, DMSO-*d*_6_, 300 K) 8.13 (s, 1H), 4.04–4.10 (m, 1H), 3.90–3.94 (s, 1H), 3.80–3.82 (s, 1H), 3.56–3.59 (m, 2H), 3.10–3.19 (m, 3H), 2.01–2.03 (m, 2H), 1.82–1.87 (m, 4H), 1.51–1.59 (m, 3H), 1.38–1.42 (m, 2H), 1.25–1.37 (m, 4H), 1.50–1.95 (m, 2H), 0.95–0.99 (m, 4H), 0.85–0.93 (m, 3H), 0.86 (d, *J* = 6.6 Hz, 6H), 0.83 (d, *J* = 6.6 Hz, 3H), 0.72 (d, *J* = 6.6 Hz, 3H). ^13^C-NMR (100 MHz, DMSOD6, 300 K) 167.1, 160.3, 157.4, 63.0, 62.7, 59.5, 55.8, 54.0, 46.5, 46.0, 42.2, 41.2, 35.3, 34.4, 33.9, 29.4, 28.3, 27.7, 25.2, 24.5, 23.7, 22.9, 22.8, 19.8, 19.5, 17.5, 14.2, 12.1, 11.7, 11.4. MS (ESI) *m*/*z* [M + H]^+^: 504.0.

LCMS, 1H, and 13C-NMR data and spectra of all compounds are included as well as the structures of all 72 compounds (TPI-2359) derived from the positional scanning deconvolution of the library TPI-1955. Stability data in mouse plasma of compound TPI 2359-47. Chemistry and Biology Experimental details (Supplementary Materials).

### 3.1. P. falciparum Culture and Antiplasmodial Activity Assay

*P. falciparum* Dd2 (chloroquine-resistant) were cultured using a modified Trager and Jensen method [42] in RPMI 1640 medium with l-glutamine (Invitrogen, Carlsbad, CA, USA) and supplemented with 25 mM HEPES, pH 7.4, 26 mM NaHCO_3_, 2% dextrose, 15 mg/L hypoxanthine, 25 mg/L gentamycin, and 0.5% Albumax II in human A^+^ erythrocytes. Cultures were incubated at 37 °C in a humidified environment of 5% CO_2_ and 95% air. The compounds were serially diluted in DMSO and were added to the *P. falciparum* culture at a 1% parasitemia and 2% hematocrit in 96-well plates. The DMSO concentration never exceeded 0.125%. Following 72-h incubation at 37 °C, the viability of the parasite was determined using a SYBR green I-based fluorescent assay which measures the DNA content of the parasite [18,19,20]. Plates were frozen at −80 °C and thawed, followed by addition of 100 µL of lysis buffer (20 mM Tris-HCl, 0.08% saponin, 5 mM EDTA, 0.8% Triton X-100, and 0.01% SYBR Green I) to each well. Following incubation in the dark for 30 min at 37 °C fluorescence emission from the wells was measured using a Synergy H4 multimode plate reader (Biotek, Winooski, VT, USA) at wavelengths 485 nM for excitation and 530 nM for emission. A reduction of fluorescent signal compared to control reflects reduced DNA content resulting from growth inhibition.

### 3.2. Cytotoxicity Assay

Different dilutions of compounds were assessed for cytotoxicity [29] in HepG2 human hepatocyte cells (2500 cells/well) in 384 well clear bottom plates (Santa Cruz Biotechnology, Dallas, TX, USA). The plates were incubated for 48 h at 37 °C in a humidified environment atmosphere (5% CO_2,_ 95% air)_._ Following the addition of 20 µL MTS ((3-(4,5-dimethylthiazol-2-yl)-5-(3-carboxymethoxyphenyl)-2-(4-sulfophenyl)-2*H*-tetrazolium), CellTiter 96^®^ Aqueous nonradioactive cell proliferation assay, Promega) reagent to each well, the plates were incubated for an additional 3 h, and cell viability was obtained through measurement of the absorbance at 490 nm using Synergy H4 plate reader (Biotek, Winooski, VT, USA).

### 3.3. Stage-Specific Inhibition Assays

*P. falciparum* Dd2 cultures were tightly synchronized employing magnetic separation of schizonts [43], followed by sorbitol lysis [44]. Synchronized cultures were exposed to the inhibitor at 5 × EC_50_ at 6-, 18-, 30-, and 42-h postinvasion. At 12-h time intervals following treatment, Giemsa-stained thin smears were prepared, and microscopically assessed to determine any inhibition of intraerthrocytic maturation. Samples were also collected at the same time for flow cytometric cell cycle analysis. Samples were fixed in 0.04% glutaraldehyde in PBS, permeabilized with 0.25% Triton X-100, treated with RNAse (50 µg/mL) and stained with 10.24 µM YOYO-1 DNA binding dye (Invitrogen) [45]. YOYO-1 is highly fluorescent when intercalated with double-stranded DNA. Flow cytometry acquisition was done in CytoFLEX flow cytometer (Beckman Coulter, Indianapolis, IN, USA) at an excitation wavelength of 488 nM and an optical filter 530/30. The data was analyzed using the Cytexpert program.

### 3.4. Mouse Plasma Stability of Compound TPI 2539-47

Whole mouse blood was centrifuged at 5000 rpm for 5 min to obtain the plasma and stored at −80 °C until time of use. One milliliter of mouse plasma was spiked to 1 mM concentration with the compound. An internal standard was prepared at 100 μg/mL concentration in 100% acetonitrile. Time points and blank samples were taken in quadruple by adding 10 μL of the plasma to 90 μL of cold acetonitrile internal standard. The zero time point was taken immediately after plasma preparation. The time point samples were taken after the designated amount of time in 37 °C water bath incubation. Samples were centrifuged at 10,000 rpm for 10 min and the supernatant was dried using a speed vacuum set to medium. The samples were reconstituted in 100 μL of 10% acetonitrile. A volume of 10 μL was used for analysis. Analysis was conducted by using high performance liquid chromatography (HPLC, 20AD Shimadzu Prominence, Kyoto, Japan)/tandem mass spectrophotometry (MS/MS, AbSciex 3200 QTrap). In order to achieve separation on the HPLC of 2359-47 and the internal standard, reverse phase mode with a gradient of 20–50% acetonitrile over 8 min was used. Mobile phase A was Fluka LCMS grade water with 0.1% formic acid. Mobile phase B was Fluka LCMS grade acetonitrile with 0.1% formic acid. The MS/MS analysis was performed in Multiple Reaction Monitoring (MRM) mode using the largest fragments of the parent compounds. The ratio of the analyte peak area to the internal standard peak area was calculated. Then, the average of the blank samples was subtracted from the average of each time point. The adjusted ratio value vs. time point was plotted.

## 4. Conclusions

In conclusion, the screening of a large complex library using a demonstrated synthetic and screening approach led to the rapid identification of antiplasmodial compounds which exhibited interesting antimalarial activity primarily against the chloroquine resistant Dd2 strain. Bis-cyclic guanidine compounds have the promise to be effective in malaria therapy. Particularly, the effect of TPI 2359-47 on schizonts is noteworthy as only artemisinins among current antimalarials demonstrate schizonticidal activities [46]. Our study underscores the utility of the positional scanning libraries in rapid identification of novel antiplasmodial hits similar to previous large-scale screening [47] of large pharmaceutical libraries. Future studies of the antiplasmodial compounds identified from this library will focus on SAR to further improve the potency.

## Supplementary Materials

The following are available online.

## Abbreviations

ESI-MS	electron spray ionization mass spectrometry
^1^H-NMR	H-nuclear magnetic resonance
^13^C-NMR	C (isotope 13)-nuclear magnetic resonance
LC-MS	liquid chromatography coupled to mass spectrometry
RP-HPLC	reverse phase high-performance liquid chromatography
TFA	trifluoroacetic acid
UV	ultraviolet
DCM	dichloromethane
THF	tetrahydrofuran
HF	hydrogen fluoride
