# Supplementary material for: Identification of Bis-Cyclic Guanidines as Antiplasmodial Compounds from Positional Scanning Mixture-Based Libraries
Source: Molecules. 2019 Mar 20;24(6):1100. doi: 10.3390/molecules24061100 (PMC6471430; doi:10.3390/molecules24061100)
Supplement: Supplementary file 1 [file molecules-24-01100-s001.pdf]

Identification of Bis Cyclic Guanidines as Antiplasmodial Compounds from Positional Scanning Mixture Based Libraries

David L. Perry II[1,ǂ], Bracken F. Roberts[1, ǂ], Ginamarie Debevec,[2]
Debopam Chakrabarti [1] and Adel Nefzi [2, *]

[1]Division of Molecular Biology and Microbiology, Burnett School of Biomedical Sciences, University of Central Florida, Orlando, FL 32826, USA

[2]Torrey Pines Institute for Molecular Studie, 11350 SW Village Parkway, Port Saint Lucie, FL 34987

Supporting Information

Pages 2-4: Building blocks used for the synthesis of library TPI-1955

Pages 5-7: Structures of all compounds derived from the deconvolution of library TPI- 1955

Pages 8-14: LCMS of reported active compounds

Pages 15-28: ^{1}H NMR and ^{13}C of reported active compounds

Page 29: Stability in mouse plasma of compound TPI 2359-47

	R1	R2	R3	R4
TPI-1955-1	Boc-Ala-OH	X	X	X
TPI-1955-2	Boc-Phe-OH	X	X	X
TPI-1955-3	Boc-Gly-OH	X	X	X
TPI-1955-4	Boc-Ile-OH·½H_2O	X	X	X
TPI-1955-5	Boc-Leu-OH·H_2O	X	X	X
TPI-1955-6	Boc-Ser(Bzl)-oh	X	X	X
TPI-1955-7	Boc-Thr(Bzl)-OH	X	X	X
TPI-1955-8	Boc-Val-OH	X	X	X
TPI-1955-9	Boc-Tyr(2-Br-Z)-OH	X	X	X
TPI-1955-10	Boc-D-Ala-OH	X	X	X
TPI-1955-11	Boc-D-Phe-OH	X	X	X
TPI-1955-12	Boc-D-Ile-OH	X	X	X
TPI-1955-13	Boc-D-Leu-OH·H_2O	X	X	X
TPI-1955-14	Boc-D-Ser(Bzl)-oh	X	X	X
TPI-1955-15	Boc-D-Thr(Bzl)-OH	X	X	X
TPI-1955-16	Boc-D-Val-OH	X	X	X
TPI-1955-17	Boc-D-Tyr(2-Br-Z)-OH	X	X	X
TPI-1955-18	Boc-Phg-OH	X	X	X
TPI-1955-19	Boc-Nva-OH	X	X	X
TPI-1955-20	Boc-D-Nva-OH	X	X	X
TPI-1955-21	Boc-Nle-OH	X	X	X
TPI-1955-22	Boc-D-Nle-OH	X	X	X
TPI-1955-23	Boc-Ala(2-naphthyl)-OH	X	X	X
TPI-1955-24	Boc-D-Ala(2-naphthyl)-OH	X	X	X
TPI-1955-25	Boc-Cha-OH	X	X	X
TPI-1955-26	Boc-D-Cha-OH	X	X	X
TPI-1955-27	X	Boc-Ala-OH	X	X
TPI-1955-28	X	Boc-Phe-OH	X	X
TPI-1955-29	X	Boc-Gly-OH	X	X
TPI-1955-30	X	Boc-Ile-OH·½H_2O	X	X
TPI-1955-31	X	Boc-Leu-OH·H_2O	X	X
TPI-1955-32	X	Boc-Ser(Bzl)-oh	X	X
TPI-1955-33	X	Boc-Thr(Bzl)-OH	X	X
TPI-1955-34	X	Boc-Val-OH	X	X
TPI-1955-35	X	Boc-Tyr(2-Br-Z)-OH	X	X
TPI-1955-36	X	Boc-D-Ala-OH	X	X
TPI-1955-37	X	Boc-D-Phe-OH	X	X
TPI-1955-38	X	Boc-D-Ile-OH	X	X
TPI-1955-39	X	Boc-D-Leu-OH·H_2O	X	X
TPI-1955-40	X	Boc-D-Ser(Bzl)-oh	X	X

TPI-1955-41	X	Boc-D-Thr(Bzl)-OH	X	X
TPI-1955-42	X	Boc-D-Val-OH	X	X
TPI-1955-43	X	Boc-D-Tyr(2-Br-Z)-OH	X	X
TPI-1955-44	X	Boc-Phg-OH	X	X
TPI-1955-45	X	Boc-Nva-OH	X	X
TPI-1955-46	X	Boc-D-Nva-OH	X	X
TPI-1955-47	X	Boc-Nle-OH	X	X
TPI-1955-48	X	Boc-D-Nle-OH	X	X
TPI-1955-49	X	Boc-Ala(2-naphthyl)-OH	X	X
TPI-1955-50	X	Boc-D-Ala(2-naphthyl)-OH	X	X
TPI-1955-51	X	Boc-Cha-OH	X	X
TPI-1955-52	X	Boc-D-Cha-OH	X	X
TPI-1955-53	X	X	Boc-Ala-OH	X
TPI-1955-54	X	X	Boc-Phe-OH	X
TPI-1955-55	X	X	Boc-Gly-OH	X
TPI-1955-56	X	X	Boc-Ile-OH·½H_2O	X
TPI-1955-57	X	X	Boc-Leu-OH·H_2O	X
TPI-1955-58	X	X	Boc-Ser(Bzl)-oh	X
TPI-1955-59	X	X	Boc-Thr(Bzl)-OH	X
TPI-1955-60	X	X	Boc-Val-OH	X
TPI-1955-61	X	X	Boc-Tyr(2-Br-Z)-OH	X
TPI-1955-62	X	X	Boc-D-Ala-OH	X
TPI-1955-63	X	X	Boc-D-Phe-OH	X
TPI-1955-64	X	X	Boc-D-Ile-OH	X
TPI-1955-65	X	X	Boc-D-Leu-OH·H_2O	X
TPI-1955-66	X	X	Boc-D-Ser(Bzl)-oh	X
TPI-1955-67	X	X	Boc-D-Thr(Bzl)-OH	X
TPI-1955-68	X	X	Boc-D-Val-OH	X
TPI-1955-69	X	X	Boc-D-Tyr(2-Br-Z)-OH	X
TPI-1955-70	X	X	Boc-Phg-OH	X
TPI-1955-71	X	X	Boc-Nva-OH	X
TPI-1955-72	X	X	Boc-D-Nva-OH	X
TPI-1955-73	X	X	Boc-Nle-OH	X
TPI-1955-74	X	X	Boc-D-Nle-OH	X
TPI-1955-75	X	X	Boc-Ala(2-naphthyl)-OH	X
TPI-1955-76	X	X	Boc-D-Ala(2-naphthyl)-OH	X
TPI-1955-77	X	X	Boc-Cha-OH	X
TPI-1955-78	X	X	Boc-D-Cha-OH	X
TPI-1955-79	X	X	X	1-phenyl-1-cyclopropanecarboxylic acid
TPI-1955-80	X	X	X	2-Phenylbutyric Acid
TPI-1955-81	X	X	X	3-Phenylbutyric Acid
TPI-1955-82	X	X	X	m-Tolylacetic acid

TPI-1955-83	X	X	X	3-Fluorophenylacetic Acid
TPI-1955-84	X	X	X	3-Bromophenylacetic Acid
TPI-1955-85	X	X	X	(α, α, α Trifluoro-m-Toly) acetic acid
TPI-1955-86	X	X	X	p-Tolylacetic acid
TPI-1955-87	X	X	X	4-Fluorophenylacetic acid
TPI-1955-88	X	X	X	3-Methoxyphenylacetic acid
TPI-1955-89	X	X	X	4-Bromophenylacetic acid
TPI-1955-90	X	X	X	4-Methoxyphenylacetic acid
TPI-1955-91	X	X	X	4-ethoxyphenylacetic acid
TPI-1955-92	X	X	X	4-isobutyl-alpha-Methylphenylacetic Acid
TPI-1955-93	X	X	X	3,4-Dichlorophenylacetic acid
TPI-1955-94	X	X	X	3,5-Bis(Trifluoromethyl)-Phenylacetic acid
TPI-1955-95	X	X	X	3-(3,4-Dimethoxyphenyl)-propionic Acid
TPI-1955-96	X	X	X	Phenylacetic acid
TPI-1955-97	X	X	X	3,4,5-Trimethoxybenzoic acid
TPI-1955-98	X	X	X	Butyric Acid
TPI-1955-99	X	X	X	Heptanoic Acid
TPI-1955-100	X	X	X	Isobutyric Acid
TPI-1955-101	X	X	X	2-Methylbutiric Acid
TPI-1955-102	X	X	X	Isovaleric acid
TPI-1955-103	X	X	X	3-Methylvaleric acid
TPI-1955-104	X	X	X	4-Methylvaleric acid
TPI-1955-105	X	X	X	p-Toluic Acid
TPI-1955-106	X	X	X	cyclopentanecarboxylic acid
TPI-1955-107	X	X	X	cyclohexanecarboxilic acid
TPI-1955-108	X	X	X	cyclohexylacetic acid
TPI-1955-109	X	X	X	cyclohexanebutyric acid
TPI-1955-110	X	X	X	cycloheptanecarboxylic acid
TPI-1955-111	X	X	X	2-Methylcyclopropanecarboxylic acid
TPI-1955-112	X	X	X	cyclobutanecarboxylic acid
TPI-1955-113	X	X	X	3-cyclopentylpropionic acid
TPI-1955-114	X	X	X	cyclohexanepropionic acid
TPI-1955-115	X	X	X	4-methyl-1-cyclohexancarboxylic acid
TPI-1955-116	X	X	X	4-tert-butyl-cyclohexancecarboxylic acid
TPI-1955-117	X	X	X	4-biphenylacetic acid
TPI-1955-118	X	X	X	1-Adamantancecarboxylic acid
TPI-1955-119	X	X	X	1-adamantaneacetic acid
TPI-1955-120	X	X	X	2-norbornaneacetic acid

2359-1
Molecular Weight: 571.68

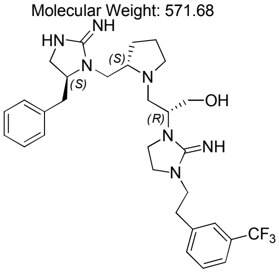

2359-2
Molecular Weight: 533.708

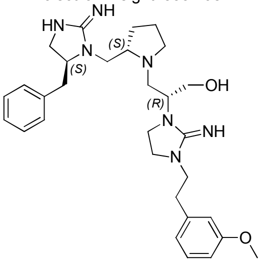

2359-3
Molecular Weight: 455.639

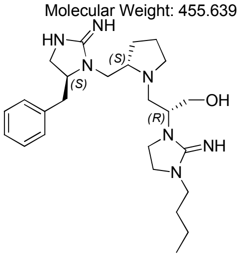

2359-4
Molecular Weight: 483.7

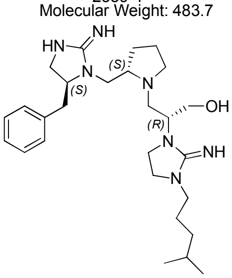

2359-5
Molecular Weight: 613.8

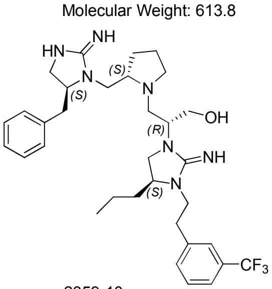

2359-6
Molecular Weight: 575.788

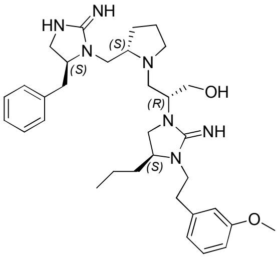

2359-7
Molecular Weight: 497.719

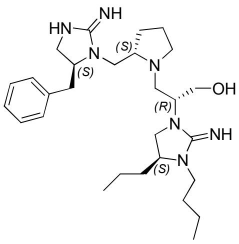

2359-8
Molecular Weight: 525.772

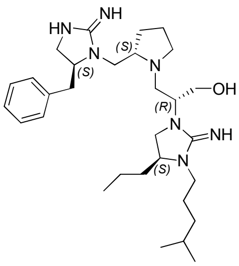

2359-9
Molecular Weight: 613.760

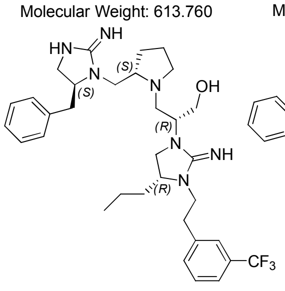

2359-10
Molecular Weight: 575.788

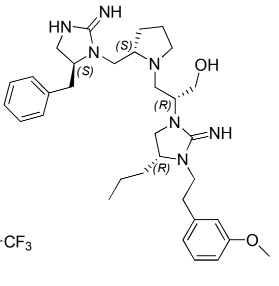

2359-11
Molecular Weight: 497.719

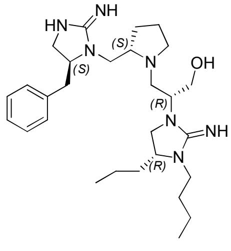

2359-12
Molecular Weight: 525.772

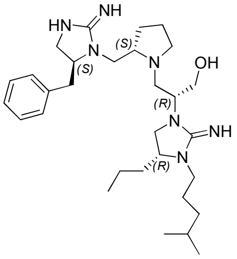

2359-13
Molecular Weight: 583.734

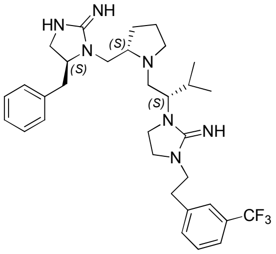

2359-14
Molecular Weight: 545.762

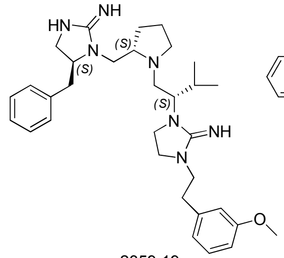

2359-15
Molecular Weight: 467.693

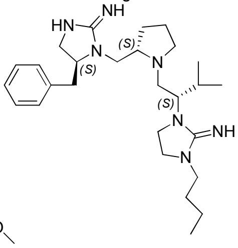

2359-16
Molecular Weight: 495.746

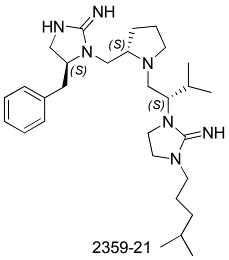

2359-17
Molecular Weight: 625.814

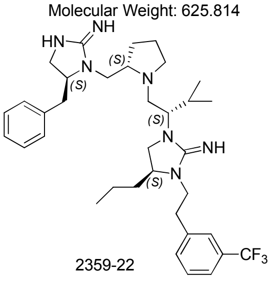

2359-18
Molecular Weight: 587.842

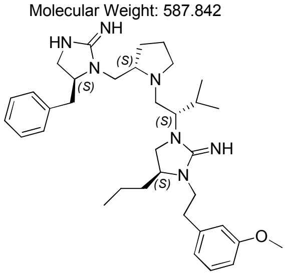

2359-19
Molecular Weight: 509.773

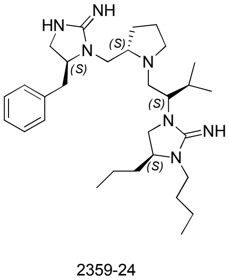

2359-20
Molecular Weight: 537.826

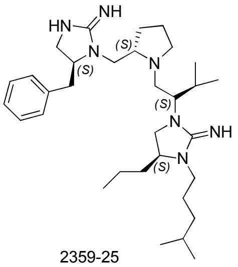

2359-21
Molecular Weight: 625.814

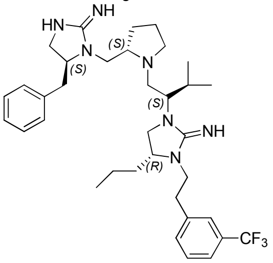

2359-22
Molecular Weight: 587.842

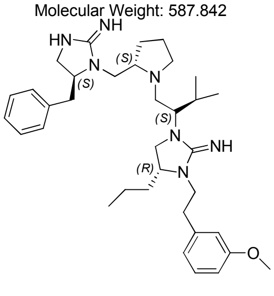

2359-23
Molecular Weight: 509.773

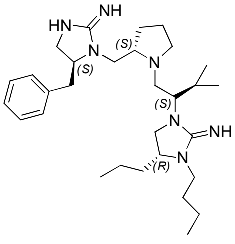

2359-24
Molecular Weight: 537.826

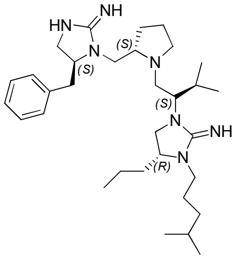

2359-25
Molecular Weight: 537.664

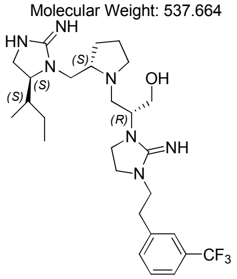

2359-26
Molecular Weight: 499.692

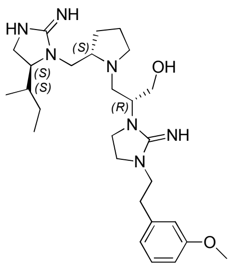

2359-27
Molecular Weight: 421.6

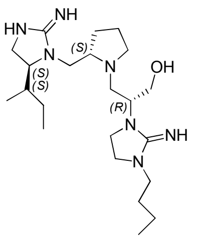

2359-28
Molecular Weight: 449.676

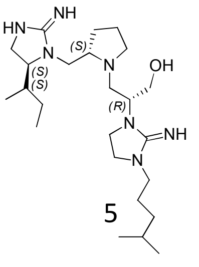

2359-29
Molecular Weight: 579.7

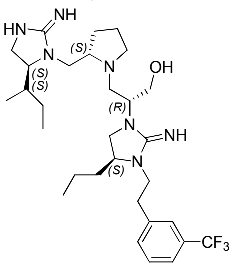

2359-30
Molecular Weight: 541.8

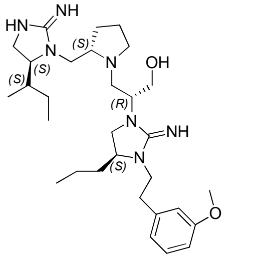

2359-31
Molecular Weight: 463.703

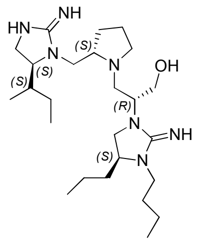

2359-32
Molecular Weight: 491.756

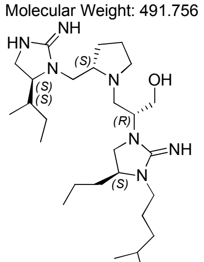

2359-33
Molecular Weight: 579.744

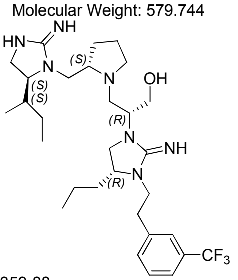

2359-34
Molecular Weight: 541.772

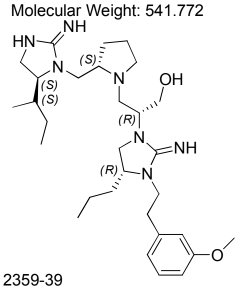

2359-35
Molecular Weight: 463.703

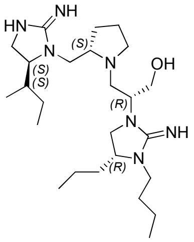

2359-36
Molecular Weight: 491.756

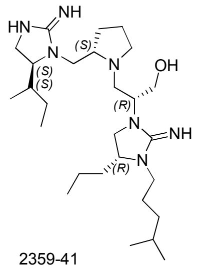

2359-37
Molecular Weight: 549.718

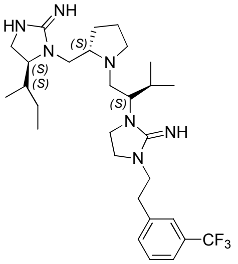

2359-38
Molecular Weight: 511.746

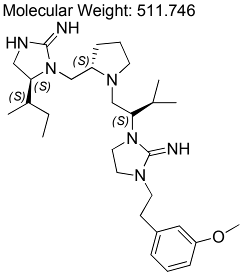

2359-39
Molecular Weight: 433.677

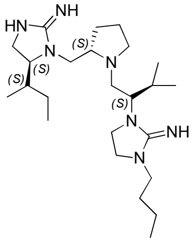

2359-40
Molecular Weight: 461.730

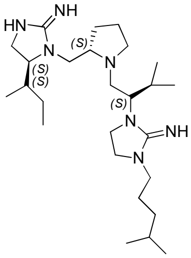

2359-41
Molecular Weight: 591.797

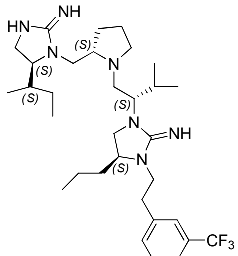

2359-42
Molecular Weight: 553.825

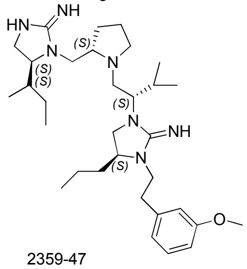

2359-43
Molecular Weight: 475.757

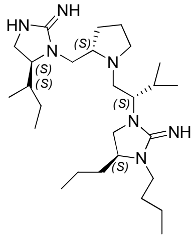

2359-44
Molecular Weight: 503.810

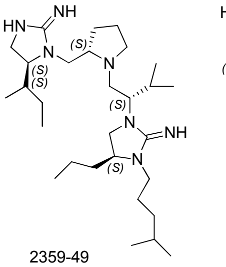

2359-45
Molecular Weight: 591.8

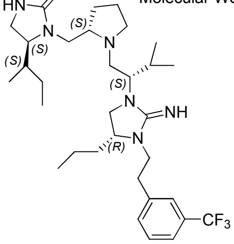

2359-46
Molecular Weight: 553.825

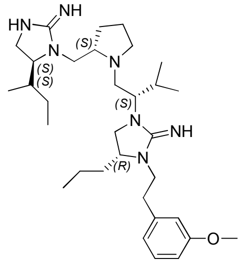

2359-47
Molecular Weight: 475.757

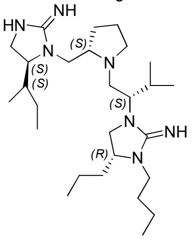

2359-48
Molecular Weight: 503.810

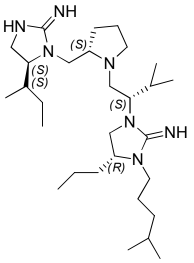

2359-49
Molecular Weight: 511.584

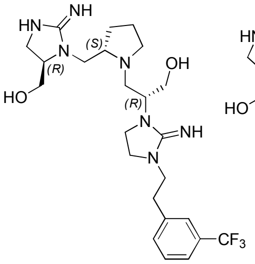

2359-50
Molecular Weight: 473.612

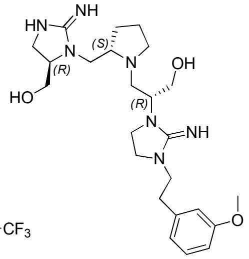

2359-51
Molecular Weight: 395.543

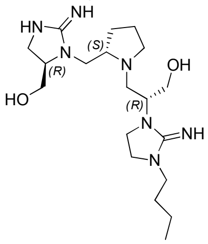

2359-52
Molecular Weight: 423.596

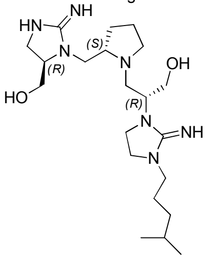

2359-53
Molecular Weight: 553.663

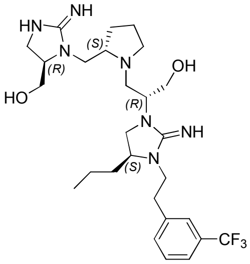

2359-54
Molecular Weight: 515.691

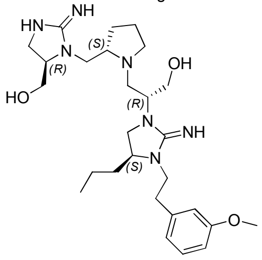

2359-55
Molecular Weight: 437.623

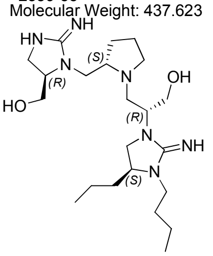

2359-56
Molecular Weight: 465.676

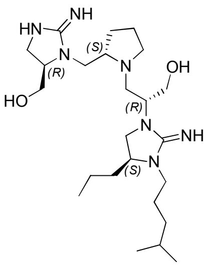

2359-57
Molecular Weight: 553.663

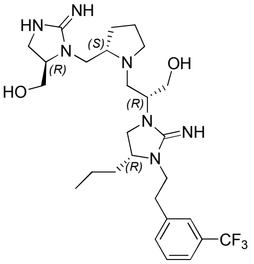

2359-58
Molecular Weight: 515.691

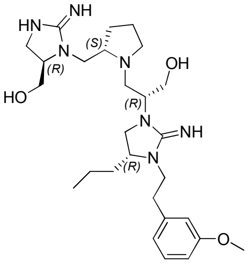

2359-59
Molecular Weight: 437.623

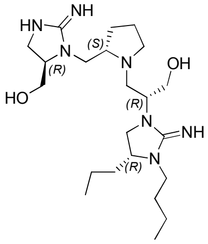

2359-60
Molecular Weight: 465.676

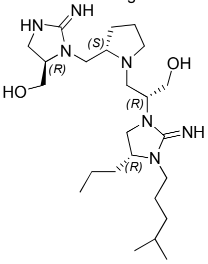

2359-61
Molecular Weight: 523.637

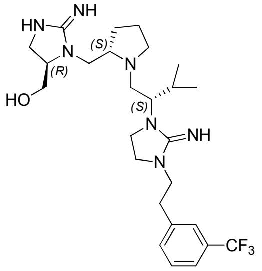

2359-62
Molecular Weight: 485.665

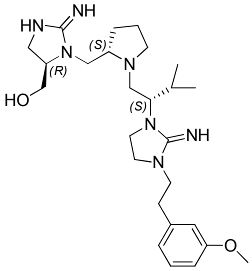

2359-63
Molecular Weight: 407.597

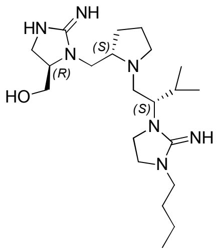

2359-64
Molecular Weight: 435.650

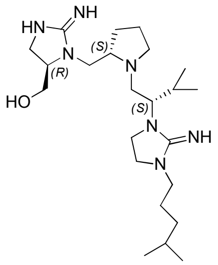

2359-65
Molecular Weight: 565.717

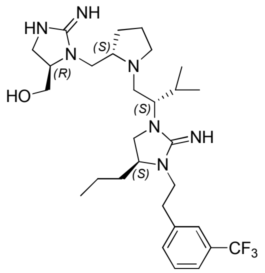

2359-66
Molecular Weight: 527.745

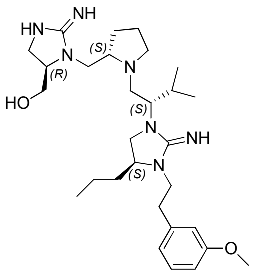

2359-67
Molecular Weight: 449.676

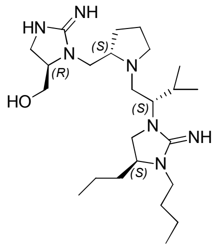

2359-68
Molecular Weight: 477.729

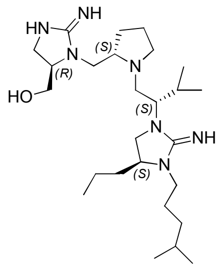

2359-69
Molecular Weight: 565.717

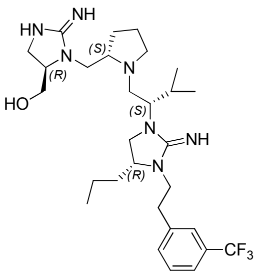

2359-70
Molecular Weight: 527.745

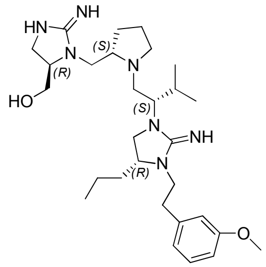

2359-71
Molecular Weight: 449.676

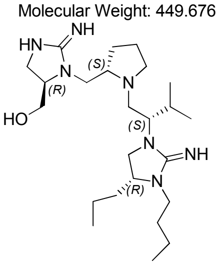

2359-72
Molecular Weight: 477.729

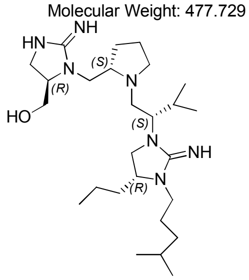

2359-30
Molecular Weight: 541.77

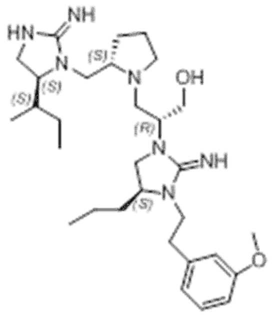

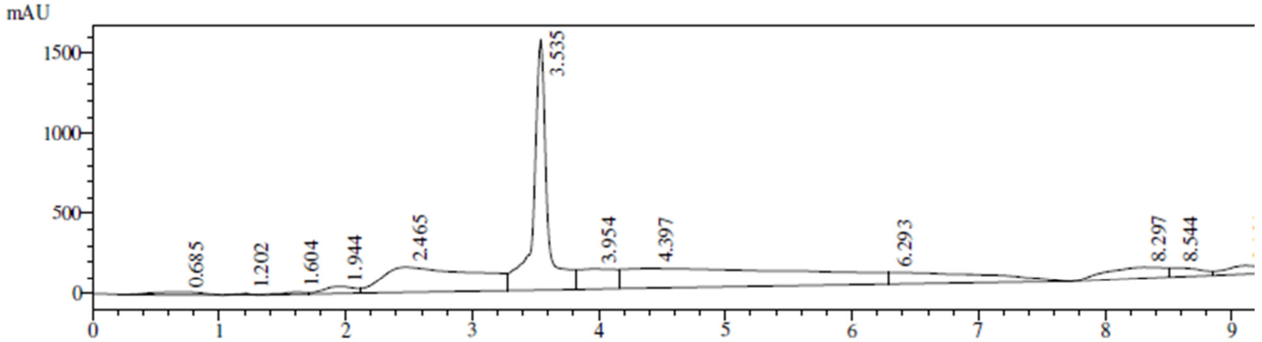

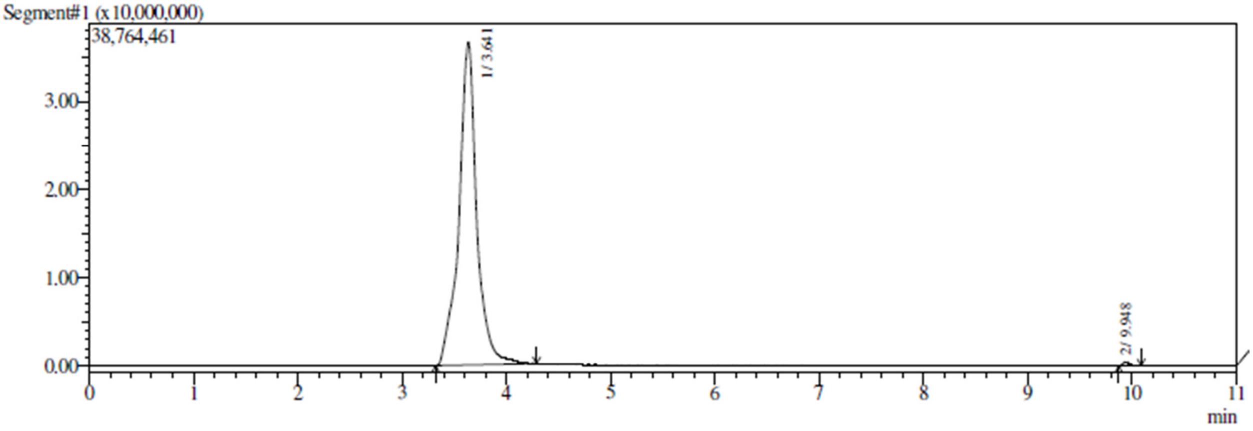

Peak#:1 Ret.Time:Averaged 3.637-3.643(Scan#:1092-1094)
BG Mode:Calc 3.320<->4.290(997<->1288)
Mass Peaks:60 Base Peak:271.75(15758717) Polarity:Pos Segment1 - Event1

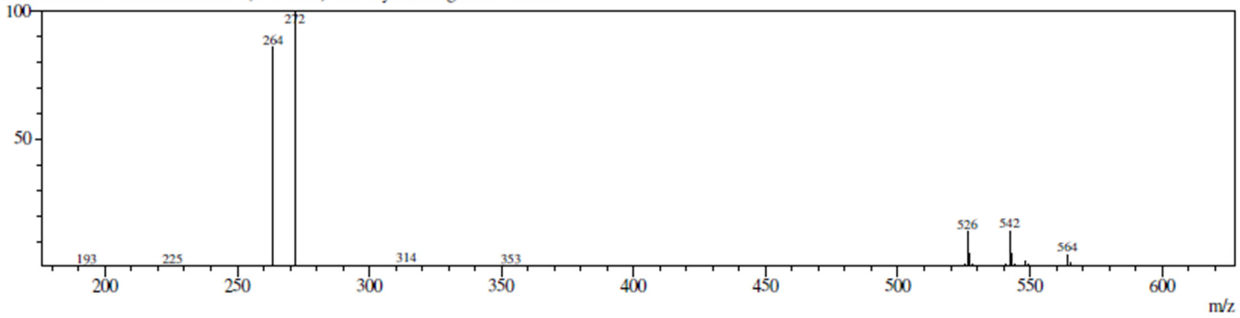

2359-31
Molecular Weight: 463.703

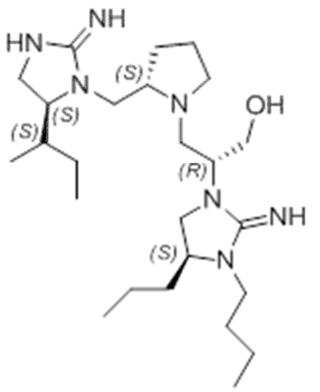

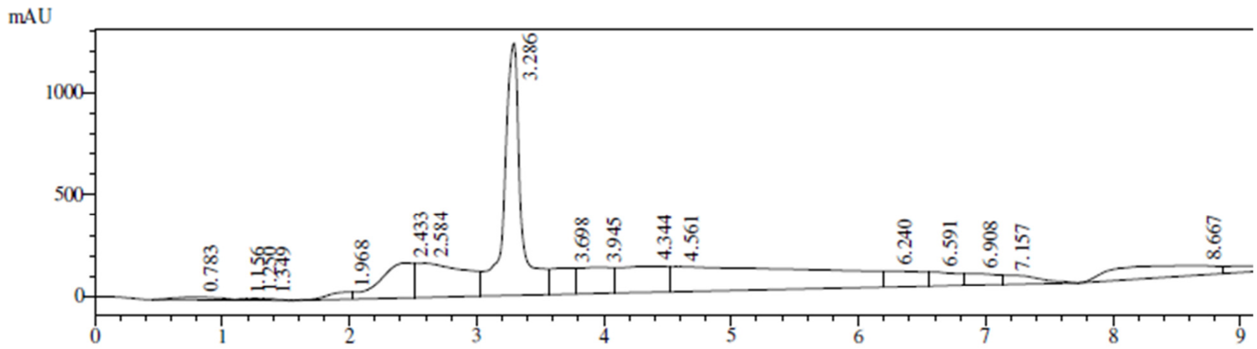

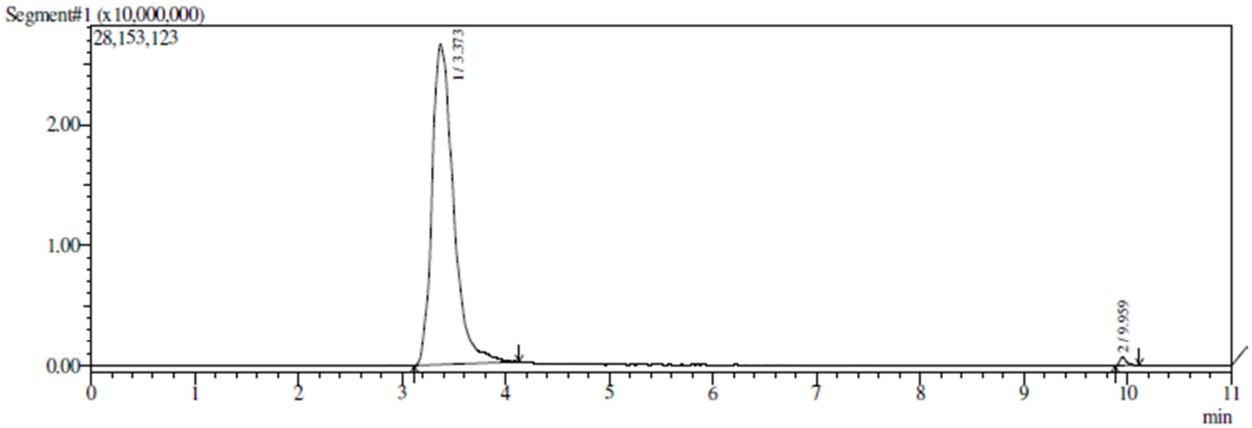

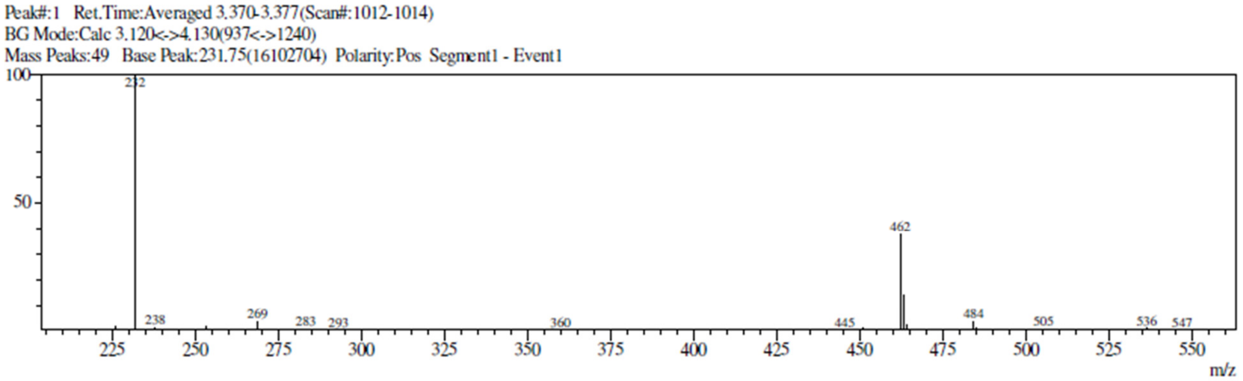

2359-32

Molecular Weight: 491.756

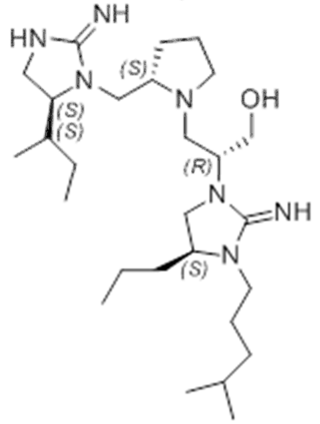

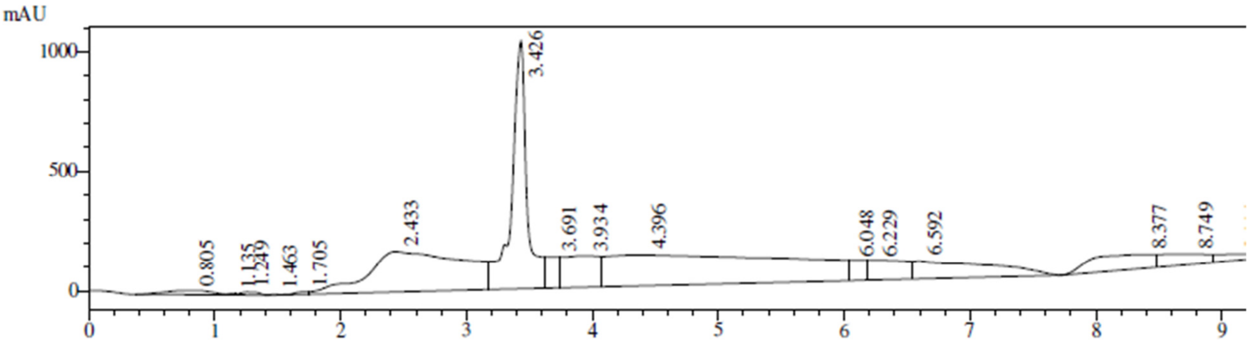

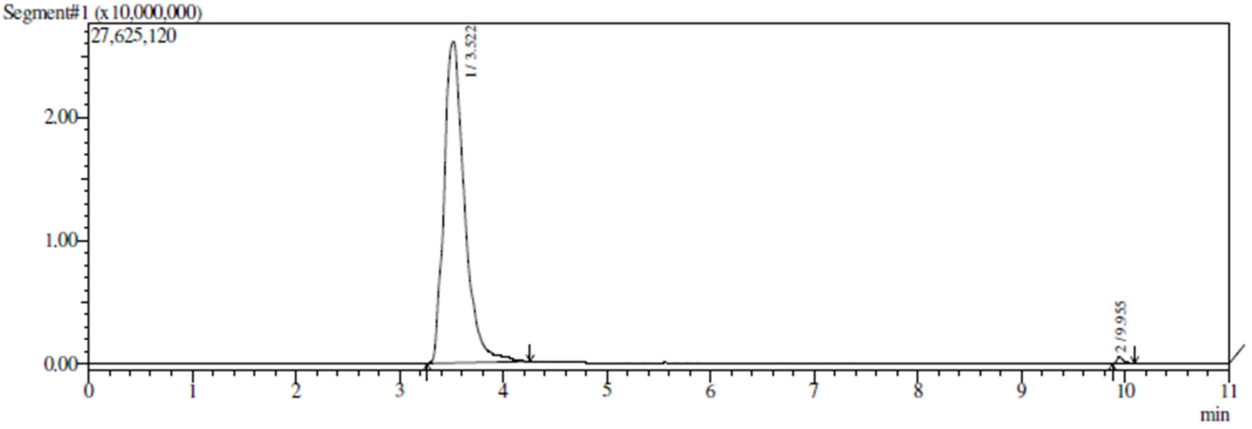

Peak#:1 Ret.Time:Averaged 3.520-3.527(Scan#:1057-1059)
BG Mode:Calc 3.267<->4.260(981<->1279)
Mass Peaks:35 Base Peak:238.75(17033442) Polarity:Pos Segment1 - Event1

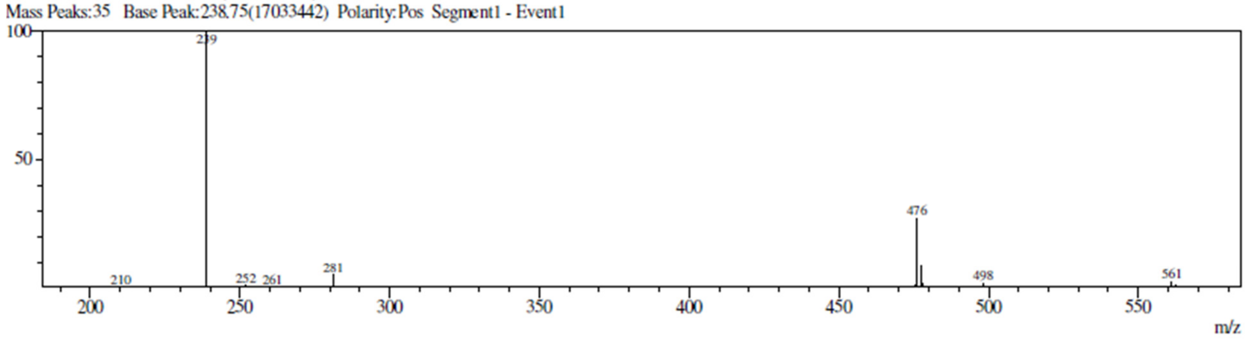

2359-40

Molecular Weight: 461.730

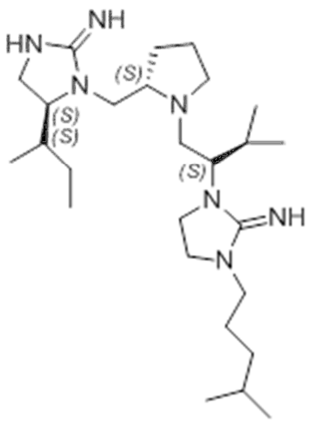

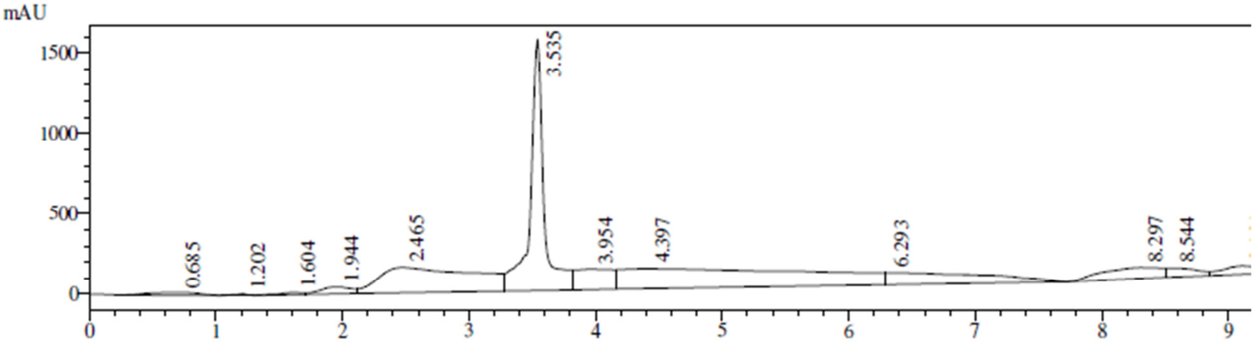

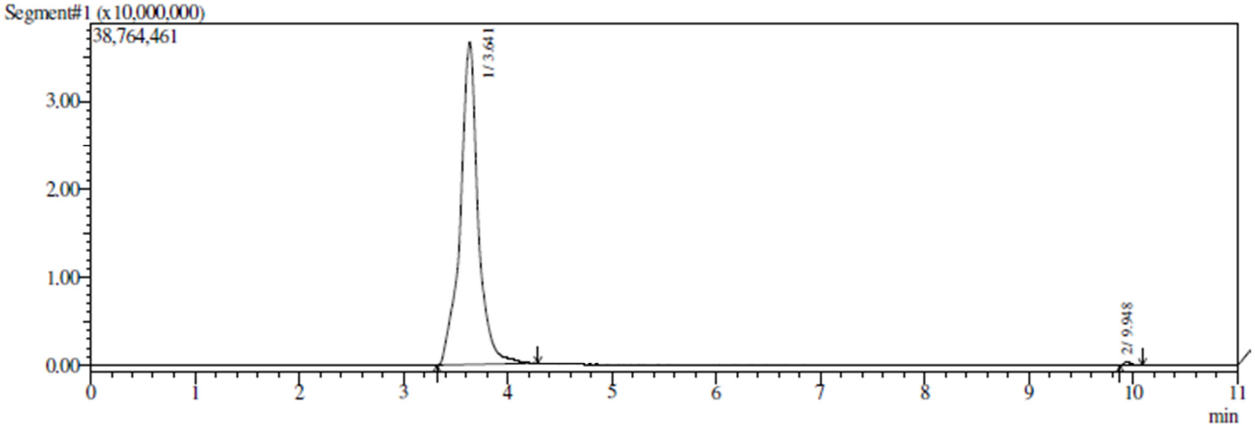

Peak#:1 Ret.Time:Averaged 3.637-3.643(Scan#:1092-1094)
BG Mode:Calc 3.320<->4.290(997<->1288)
Mass Peaks:60 Base Peak:271.75(15758717) Polarity:Pos Segment1 - Event1

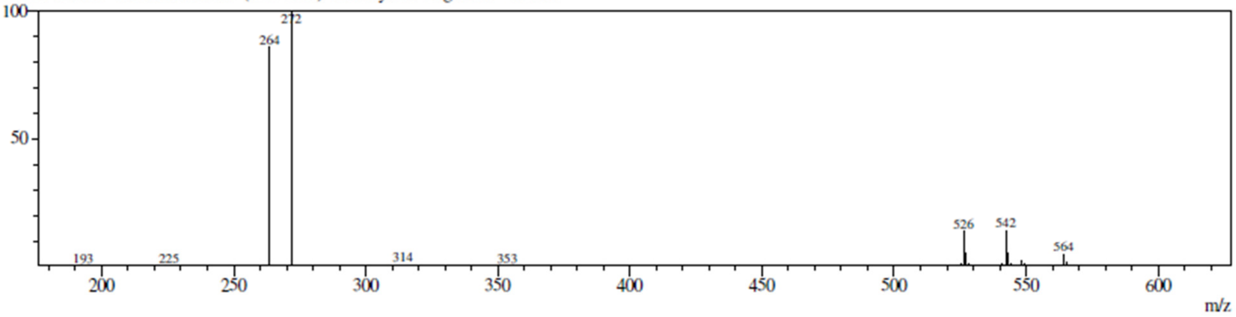

2359-45
Molecular Weight: 591.80

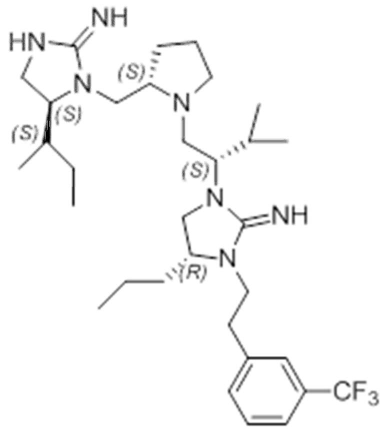

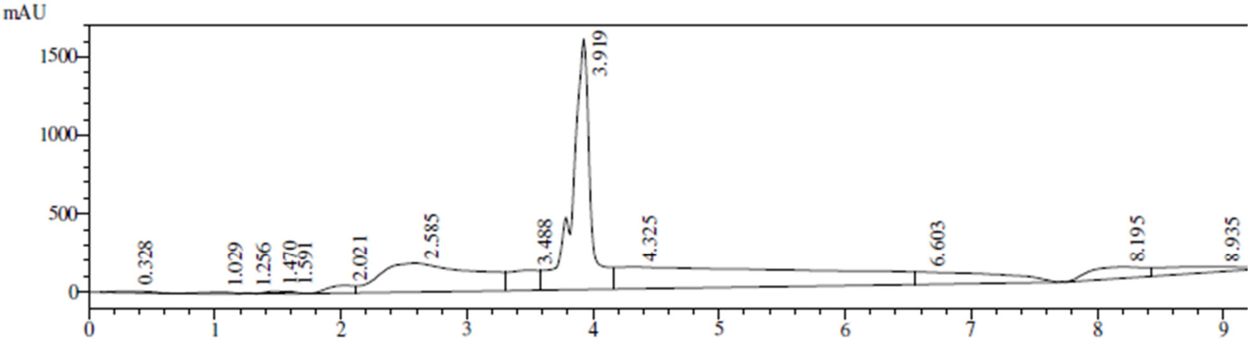

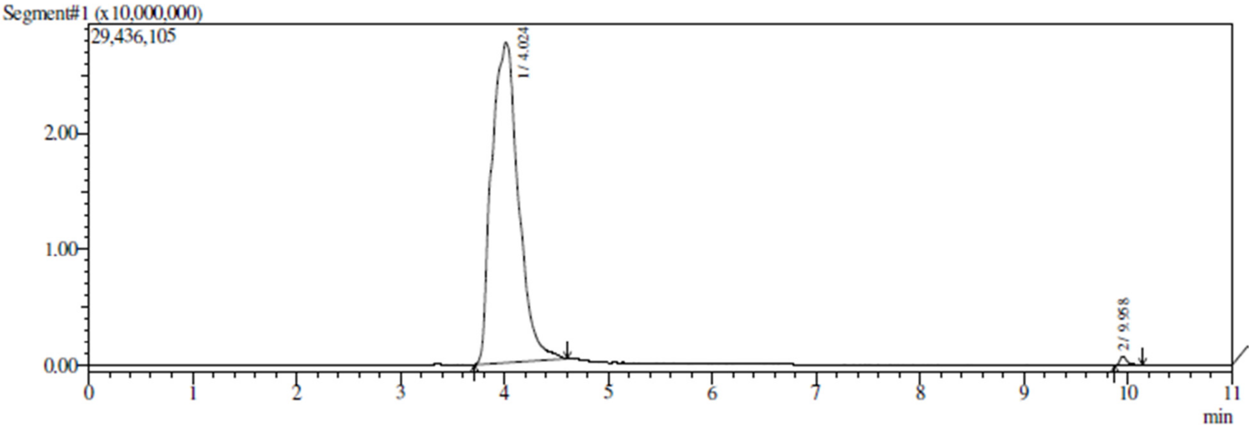

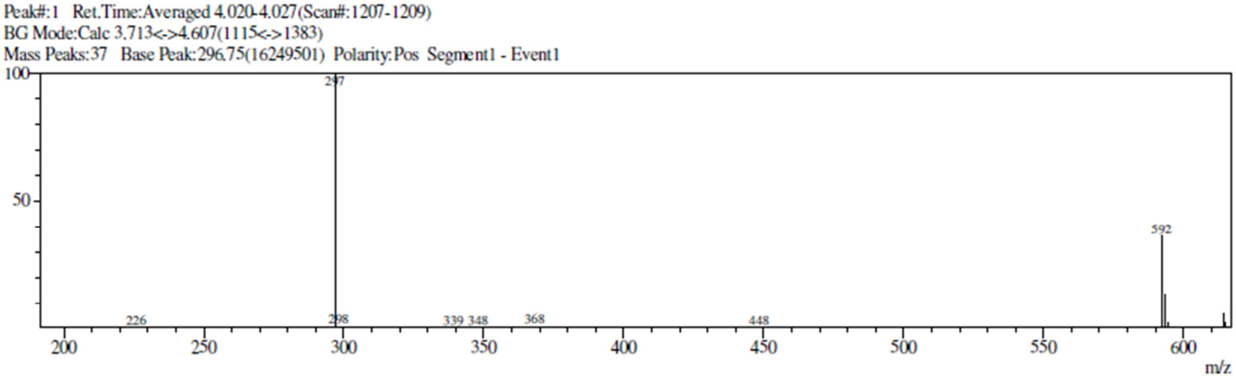

2359-47

Molecular Weight: 475.757

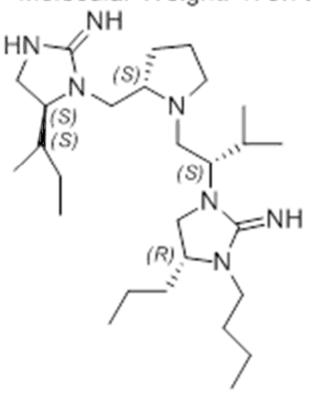

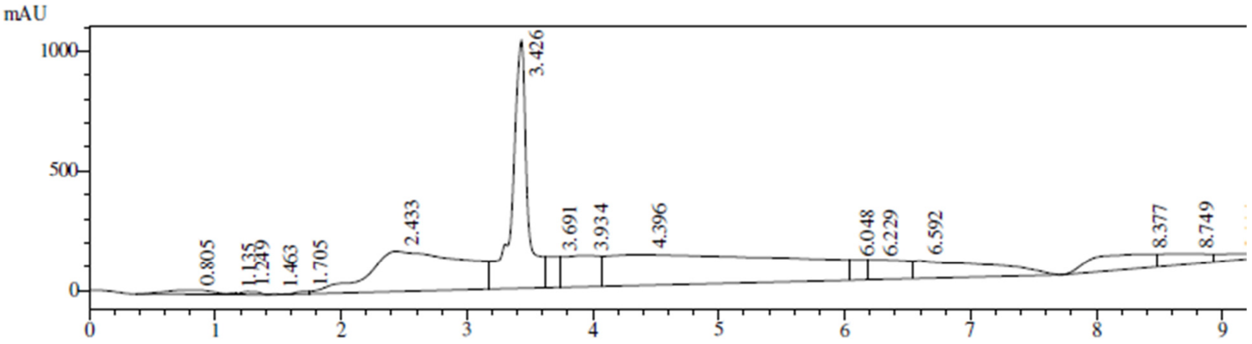

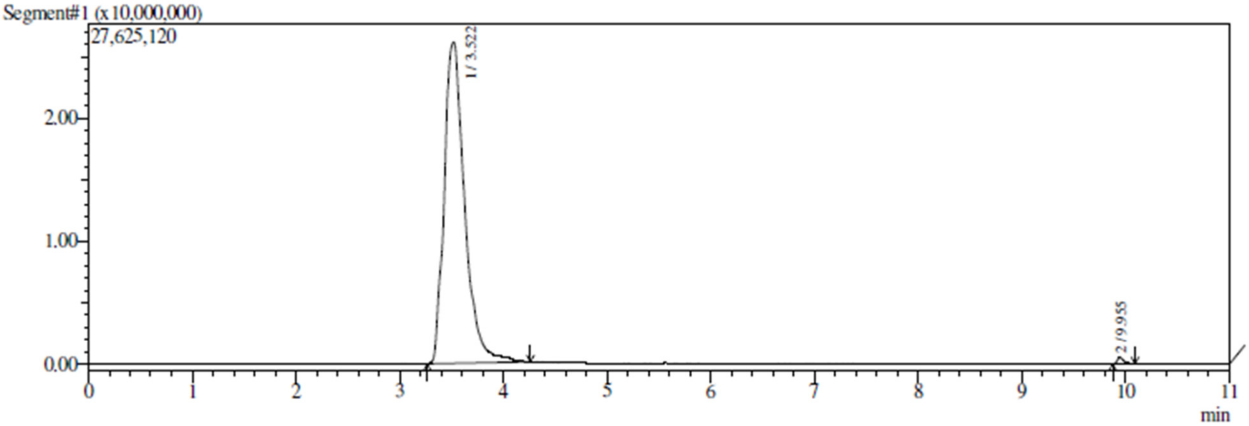

Peak#:1 Ret.Time:Averaged 3.520-3.527(Scan#:1057-1059)
BG Mode:Calc 3.267<->4.260(981<->1279)
Mass Peaks:35 Base Peak:238.75(17033442) Polarity:Pos Segment1 - Event1

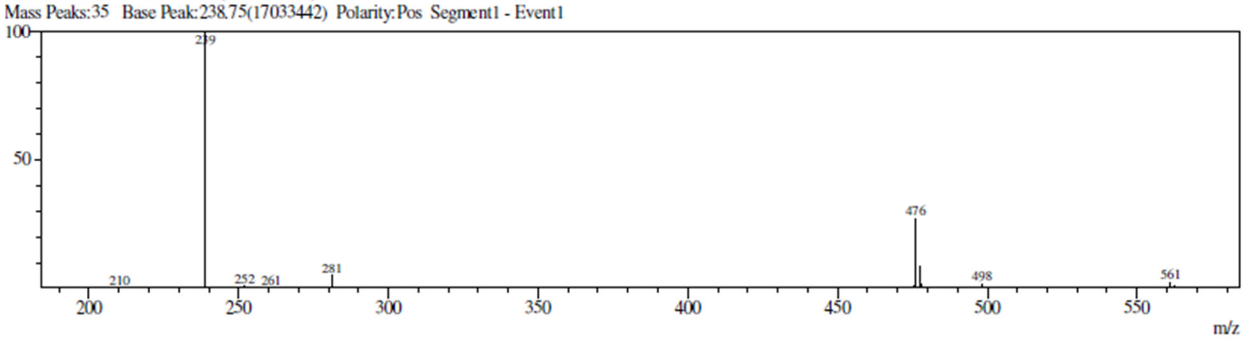

2359-48
Molecular Weight: 503.810

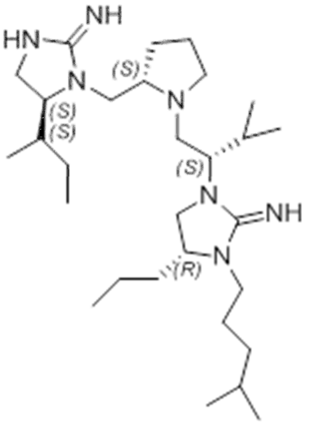

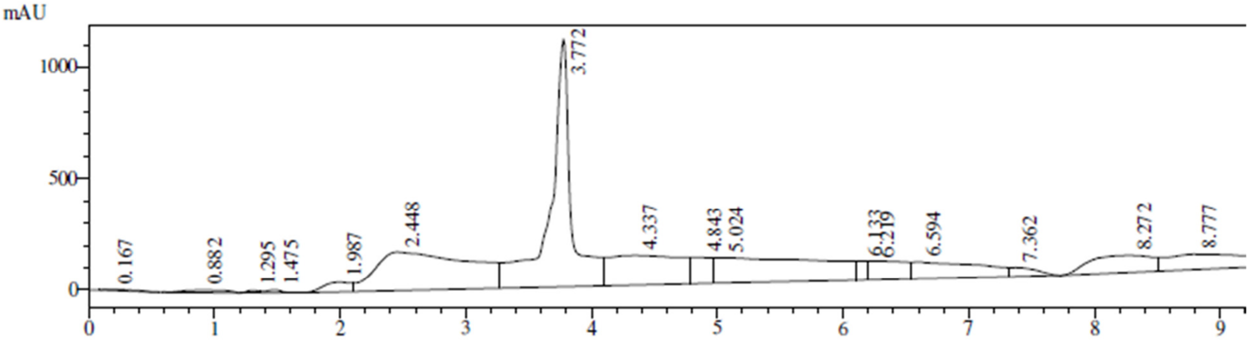

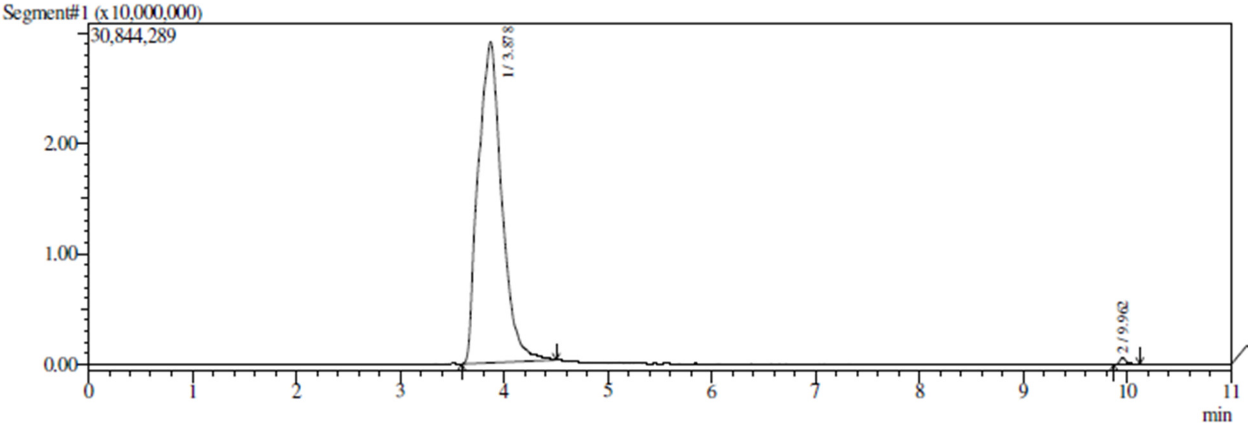

Peak#:1 Ret.Time:Averaged 3.873-3.880(Scan#:1163-1165)
BG Mode:Calc 3.593<->4.503(1079<->1352)
Mass Peaks:37 Base Peak:252.80(18632080) Polarity:Pos Segment1 - Event1

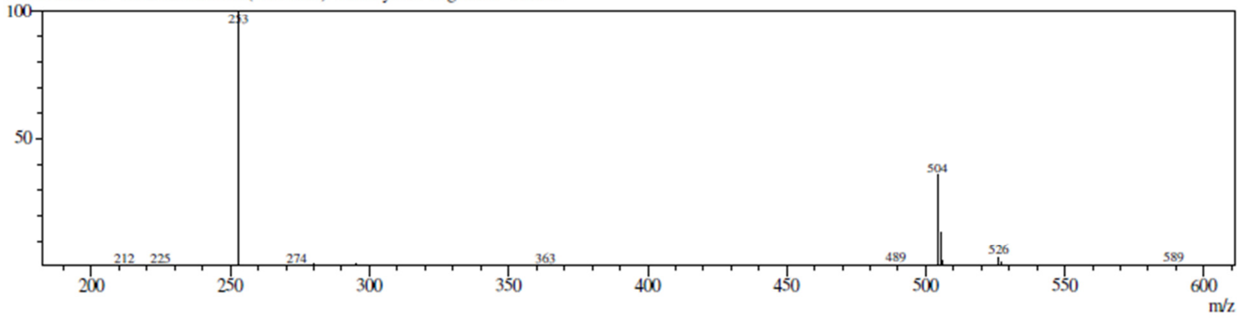

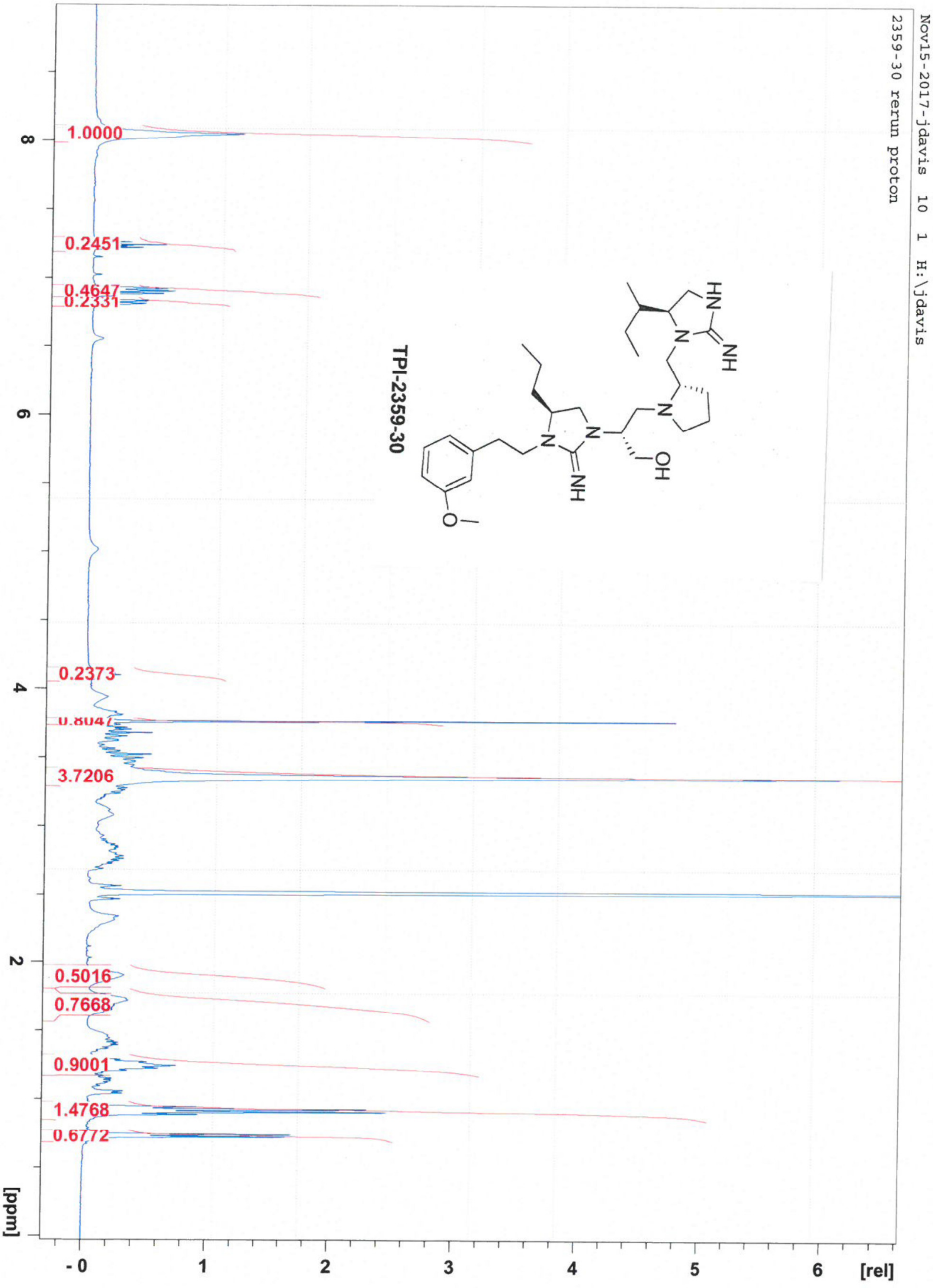

Nov15-2017-jdavis 10 1 H:\jdavis
2359-30 rerun proton
TPI-2359-30
NH
HN
N
OH
O
1.0000
0.2451
0.4647
0.2331
0.2373
0.8047
3.7206
0.5016
0.7668
0.9001
1.4768
0.6772
8
6
4
2
[ppm]
-0
1
2
3
4
5
6
[rel]

2359-30 pure C13

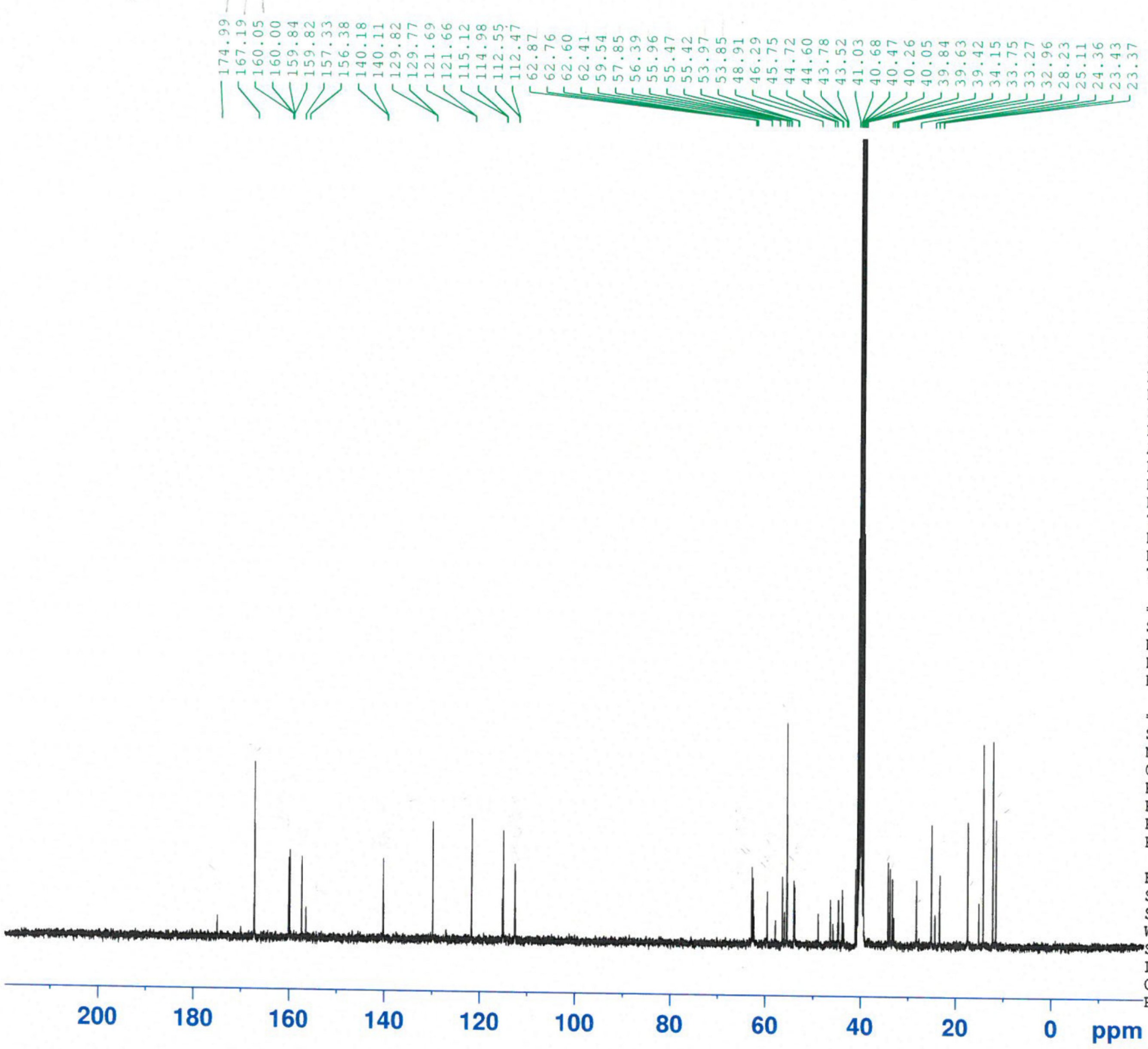

Current Data Parameters

NAME	Oct23-2017-jdavis
EXPNO	21
PROCNO	1

F2 - Acquisition Parameters

Date_	20171023
Time	16.26
INSTRUM	spect
PROBHD	5 mm PABBO BB/
PULPROG	zgpg30
TD	65536
SOLVENT	DMSO
NS	2048
DS	4
SWH	24038.461 Hz
FIDRES	0.366798 Hz
AQ	1.3631488 sec
RG	208.24
DW	20.800 usec
DE	6.50 usec
TE	299.6 K
D1	2.00000000 sec
D11	0.03000000 sec
TD0	1

======== CHANNEL f1 ========

SFO1	100.6278588 MHz
NUC1	13C
P1	10.00 usec
PLW1	64.00000000 W

======== CHANNEL f2 ========

SFO2	400.1516006 MHz
NUC2	1H
CPDPRG[2	waltz16
PCPD2	90.00 usec
PLW2	29.00000000 W
PLW12	0.35802001 W
PLW13	0.28999999 W

F2 - Processing parameters

SI	32768
SF	100.6177980 MHz
WDW	EM
SSB	0
LB	1.00 Hz
GB	0
PC	1.40

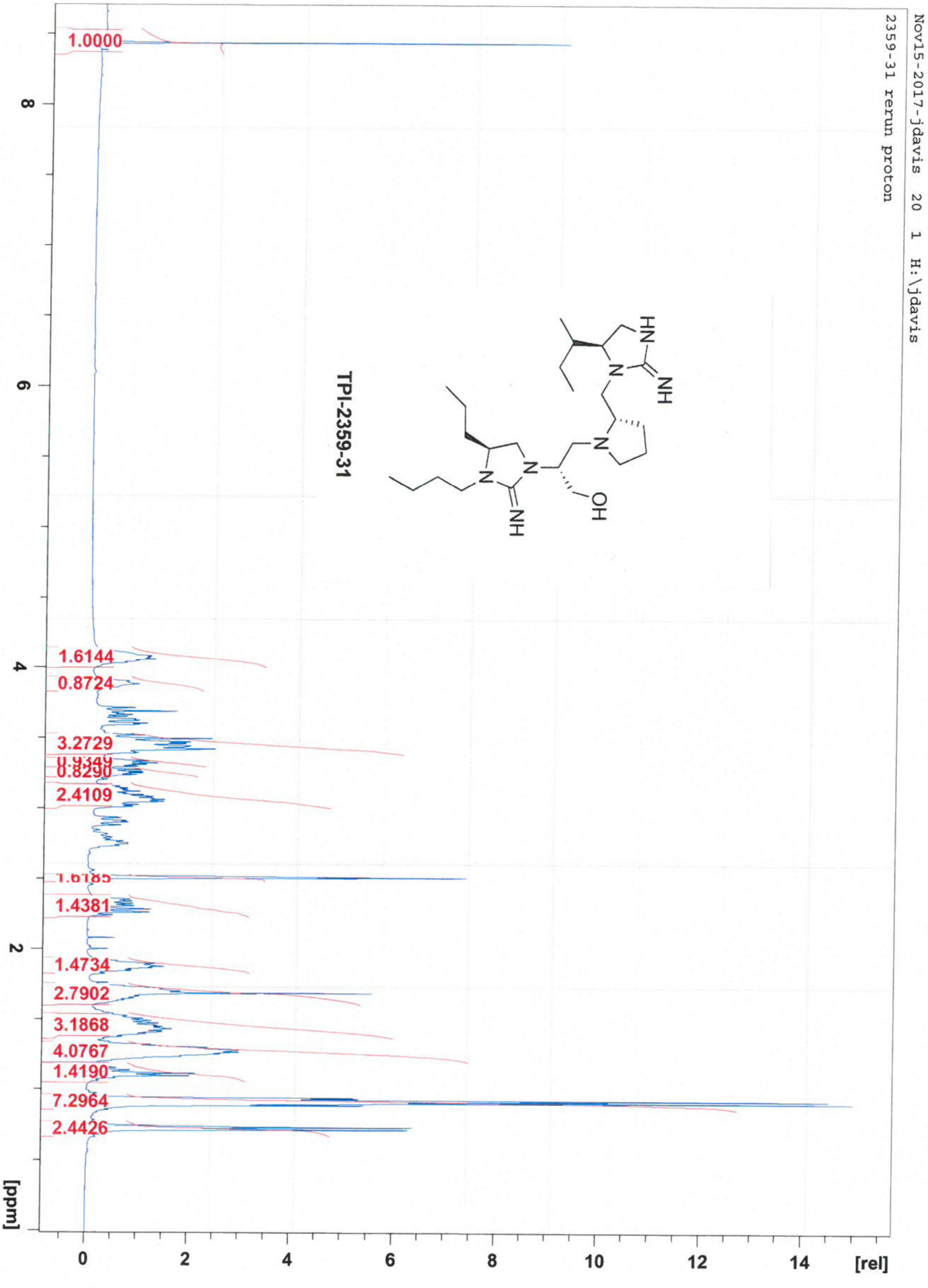

Nov15-2017-jdavis 20 1 H:\jdavis
2359-31 rerun proton
TPI-2359-31
1.0000
1.6144
0.8724
3.2729
0.8290
2.4109
1.4381
1.4734
2.7902
3.1868
4.0767
1.4190
7.2964
2.4426
[ppm]
[rel]

2359-31 pure C13

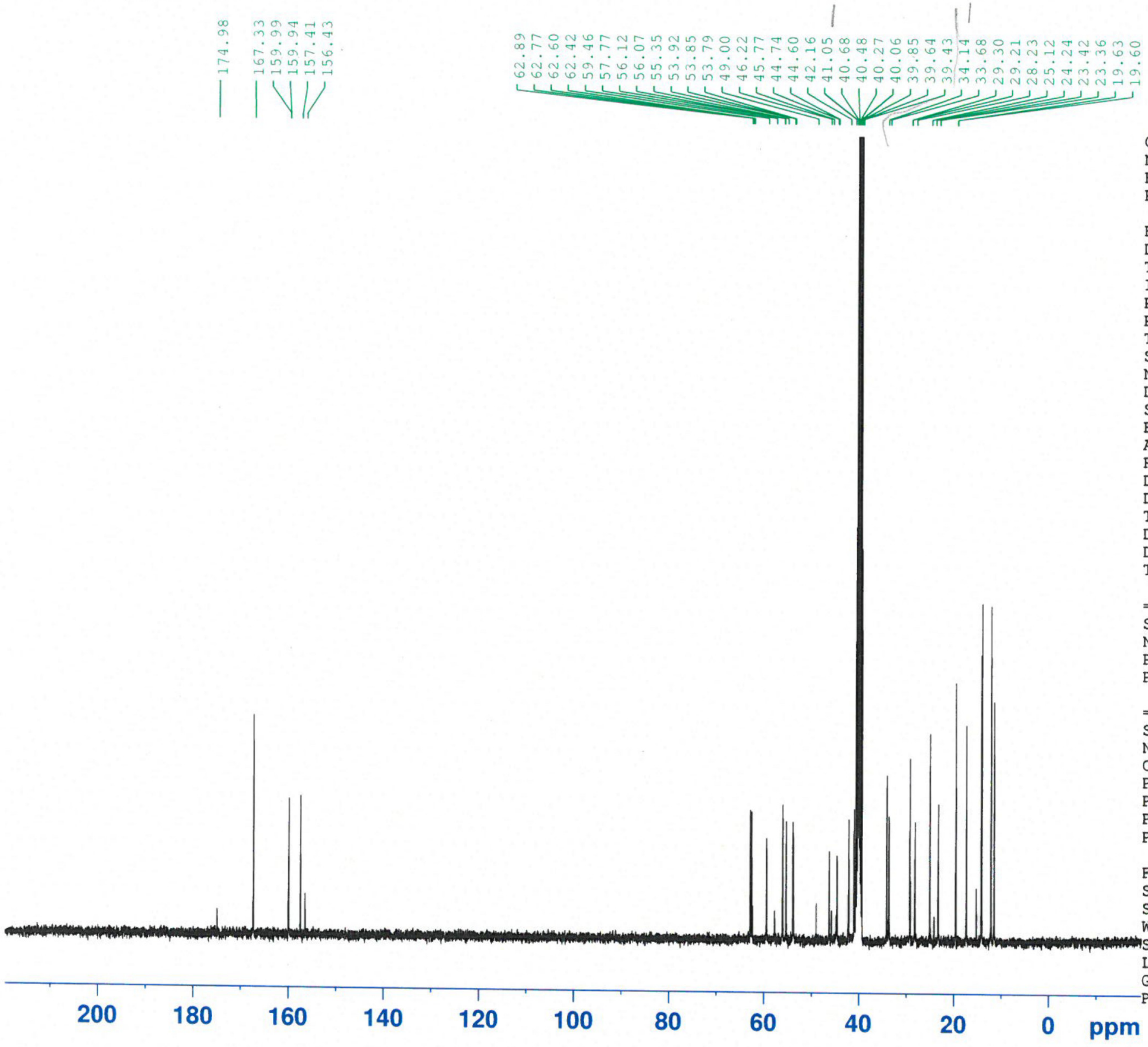


```
Current Data Parameters
NAME        Oct23-2017-jdavis
EXPNO                      31
PROCNO                      1

F2 - Acquisition Parameters
Date_                20171023
Time                    18.51
INSTRUM                 spect
PROBHD      5 mm PABBO BB/
PULPROG               zgpg30
TD                      65536
SOLVENT                  DMSO
NS                       2048
DS                          4
SWH                 24038.461 Hz
FIDRES               0.366798 Hz
AQ                  1.3631488 sec
RG                     208.24
DW                     20.800 usec
DE                       6.50 usec
TE                      299.6 K
D1                 2.00000000 sec
D11                0.03000000 sec
TD0                         1

======== CHANNEL f1 ========
SFO1              100.6278588 MHz
NUC1                      13C
P1                      10.00 usec
PLW1              64.00000000 W

======== CHANNEL f2 ========
SFO2              400.1516006 MHz
NUC2                       1H
CPDPRG[2              waltz16
PCPD2                   90.00 usec
PLW2              29.00000000 W
PLW12              0.35802001 W
PLW13              0.28999999 W

F2 - Processing parameters
SI                      32768
SF                100.6177980 MHz
WDW                        EM
SSB         0
LB                       1.00 Hz
GB          0
PC                       1.40
```

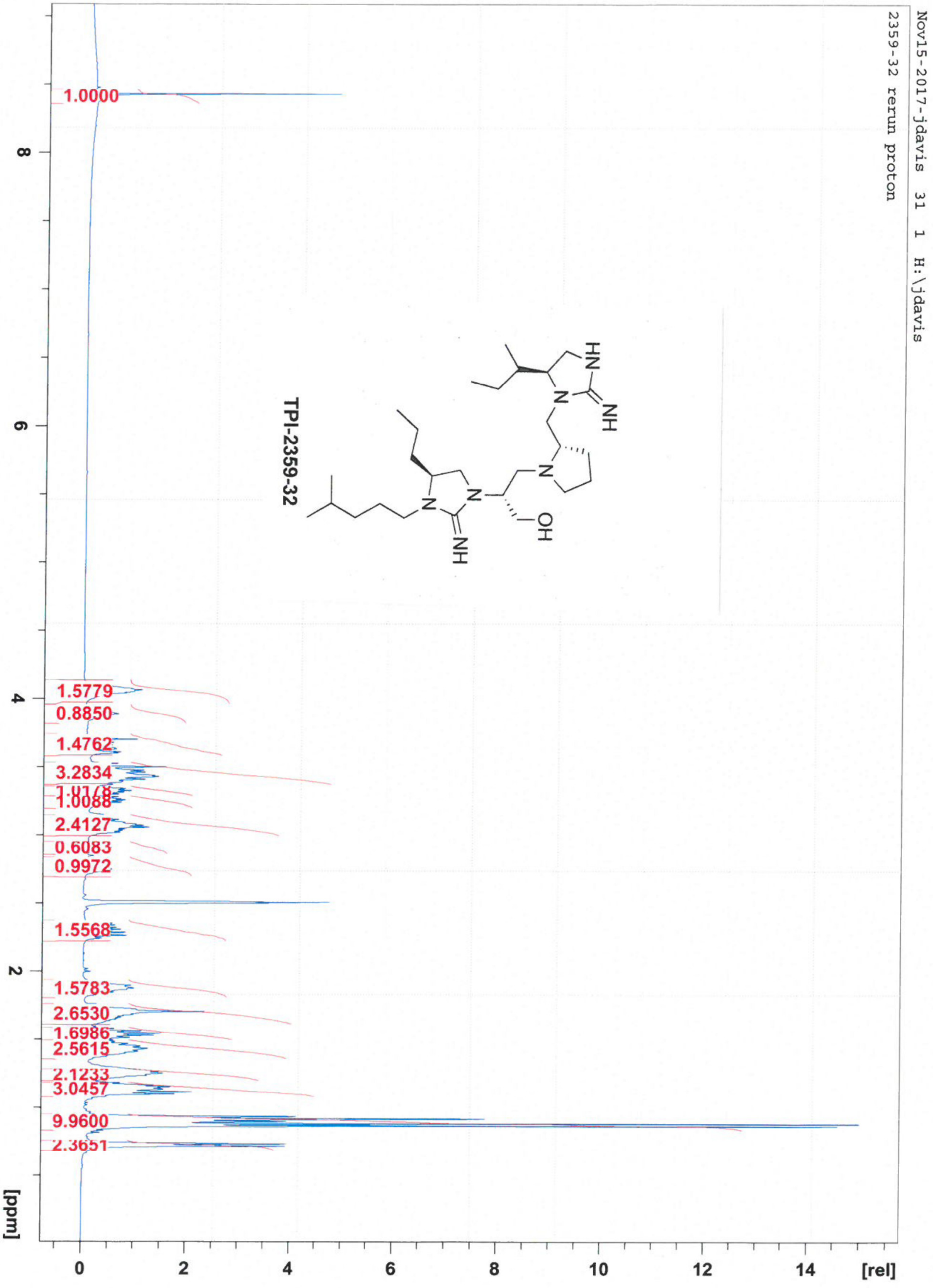
Nov15-2017-jdavis 31 1 H:\jdavis
2359-32 rerun proton
TPI-2359-32
[ppm]
[rel]

2359-32 pure C13

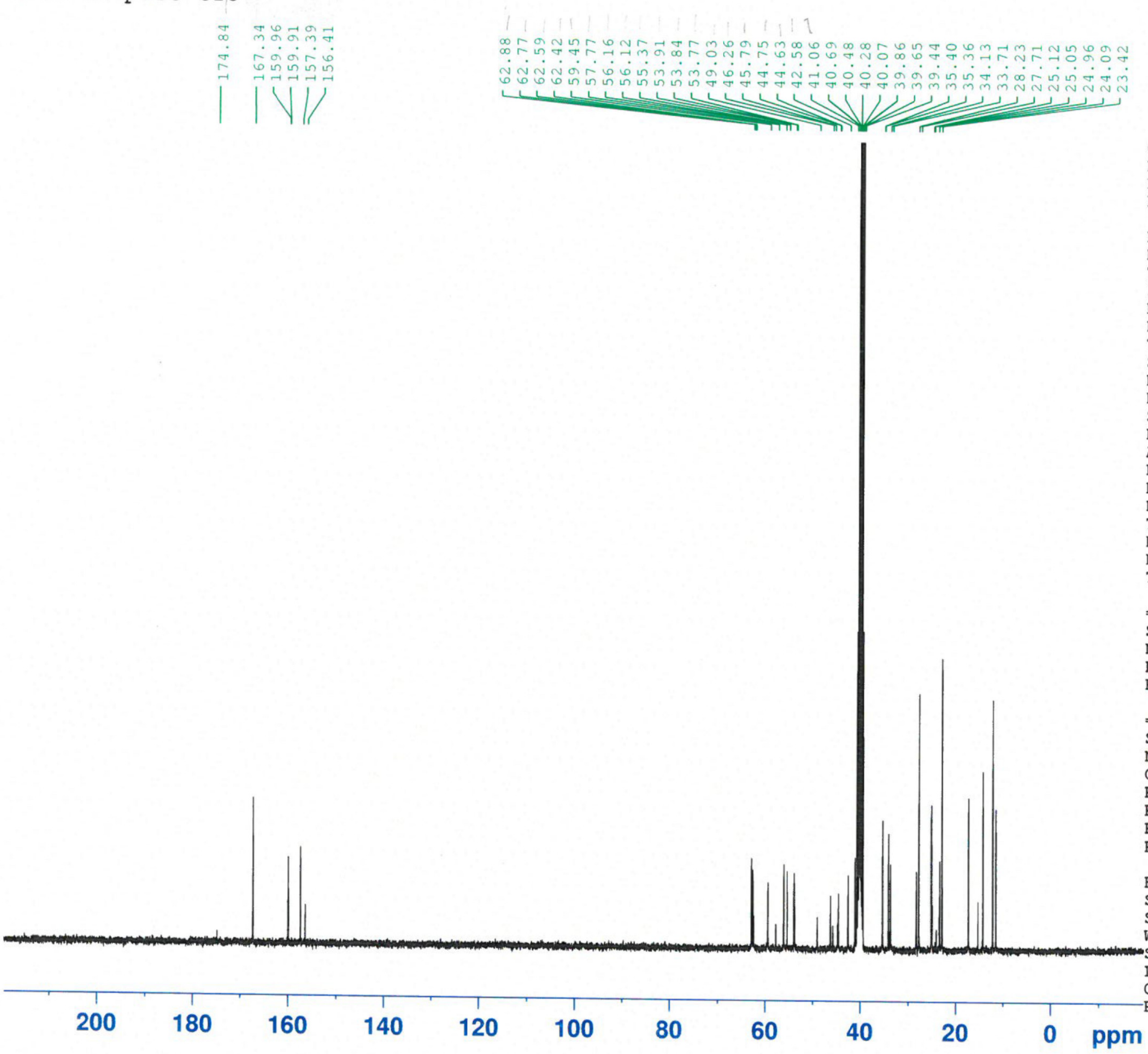

Current Data Parameters

NAME	Oct23-2017-jdavis
EXPNO	41
PROCNO	1

F2 - Acquisition Parameters

Date_	20171023
Time	21.16
INSTRUM	spect
PROBHD	5 mm PABBO BB/
PULPROG	zgpg30
TD	65536
SOLVENT	DMSO
NS	2048
DS	4
SWH	24038.461 Hz
FIDRES	0.366798 Hz
AQ	1.3631488 sec
RG	208.24
DW	20.800 usec
DE	6.50 usec
TE	299.7 K
D1	2.00000000 sec
D11	0.03000000 sec
TD0	1

======== CHANNEL f1 ========

SFO1	100.6278588 MHz
NUC1	13C
P1	10.00 usec
PLW1	64.00000000 W

======== CHANNEL f2 ========

SFO2	400.1516006 MHz
NUC2	1H
CPDPRG[2	waltz16
PCPD2	90.00 usec
PLW2	29.00000000 W
PLW12	0.35802001 W
PLW13	0.28999999 W

F2 - Processing parameters

SI		32768
SF		100.6177980 MHz
WDW		EM
SSB	0	
LB		1.00 Hz
GB	0	
PC		1.40

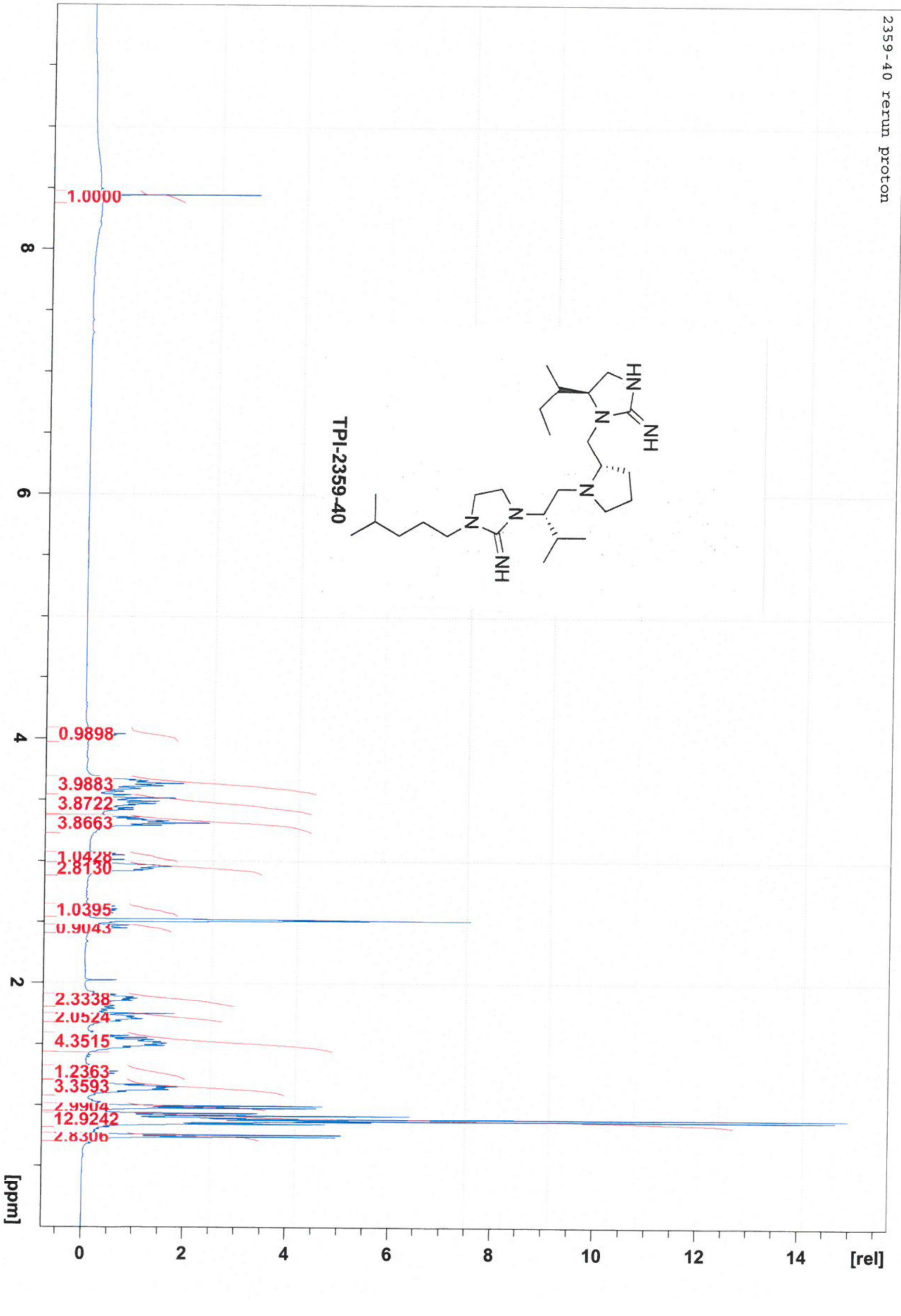
Nov15-2017-jdavis 41 1 H:\jdavis
2359-40 rerun proton
TPI-2359-40
1.0000
0.9898
3.9883
3.8722
3.8663
1.0428
2.8130
1.0395
0.9043
2.3338
2.0524
4.3515
1.2363
3.3593
2.9904
12.9242
2.8306
[ppm]
[rel]

2539-40 pure C13

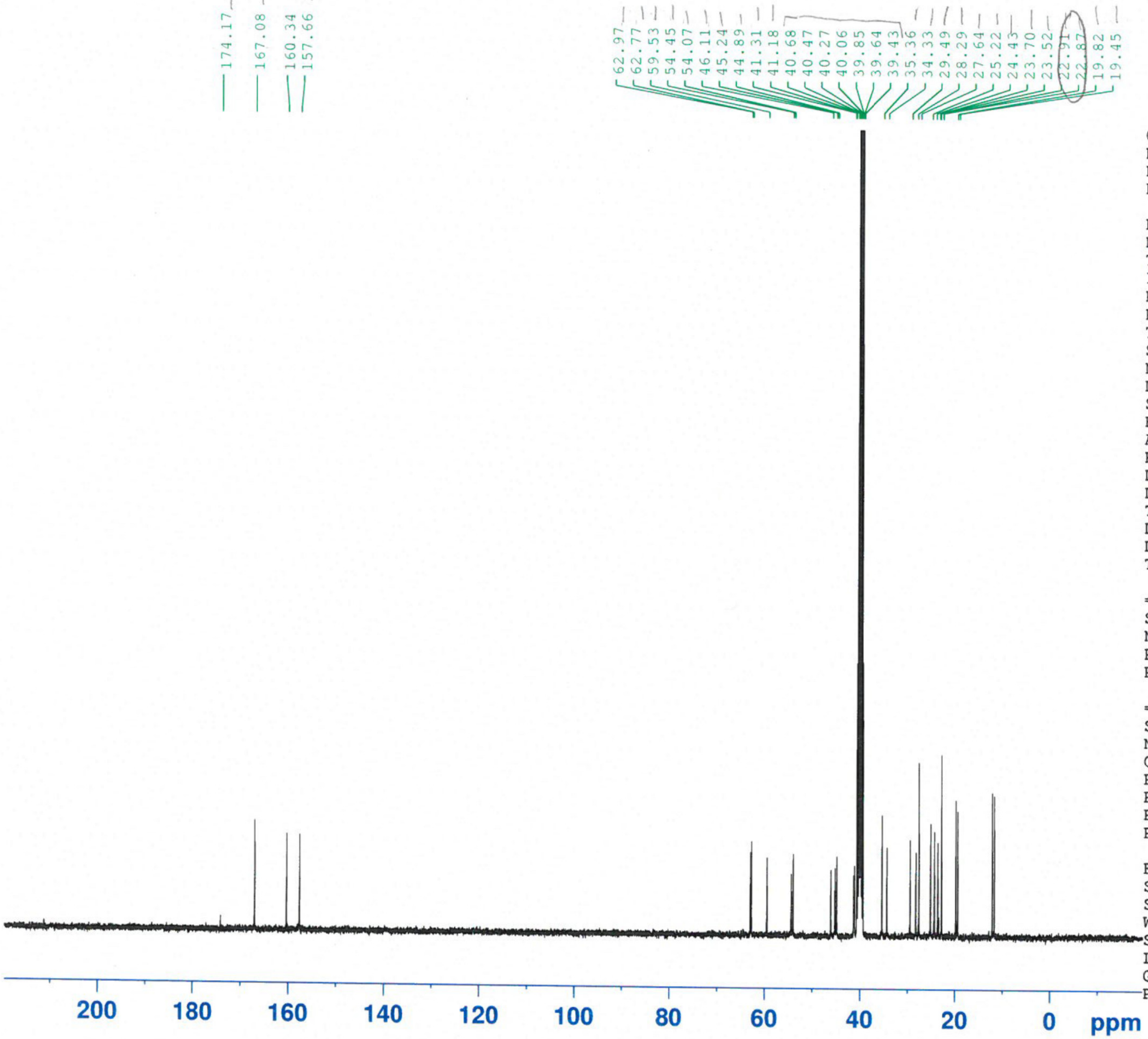


```
Current Data Parameters
NAME        Oct23-2017-jdavis
EXPNO                      51
PROCNO                      1

F2 - Acquisition Parameters
Date_                20171023
Time                    23.50
INSTRUM                 spect
PROBHD      5 mm PABBO BB/
PULPROG               zgpg30
TD                      65536
SOLVENT                  DMSO
NS                       2048
DS                          4
SWH                 24038.461 Hz
FIDRES               0.366798 Hz
AQ                  1.3631488 sec
RG                     208.24
DW                     20.800 usec
DE                       6.50 usec
TE                      299.7 K
D1                 2.00000000 sec
D11                0.03000000 sec
TD0                         1

======== CHANNEL f1 ========
SFO1              100.6278588 MHz
NUC1                      13C
P1                      10.00 usec
PLW1              64.00000000 W

======== CHANNEL f2 ========
SFO2              400.1516006 MHz
NUC2                       1H
CPDPRG[2              waltz16
PCPD2                   90.00 usec
PLW2              29.00000000 W
PLW12              0.35802001 W
PLW13              0.28999999 W

F2 - Processing parameters
SI                      32768
SF                100.6177980 MHz
WDW                        EM
SSB      0
LB                       1.00 Hz
GB       0
PC                       1.40
```

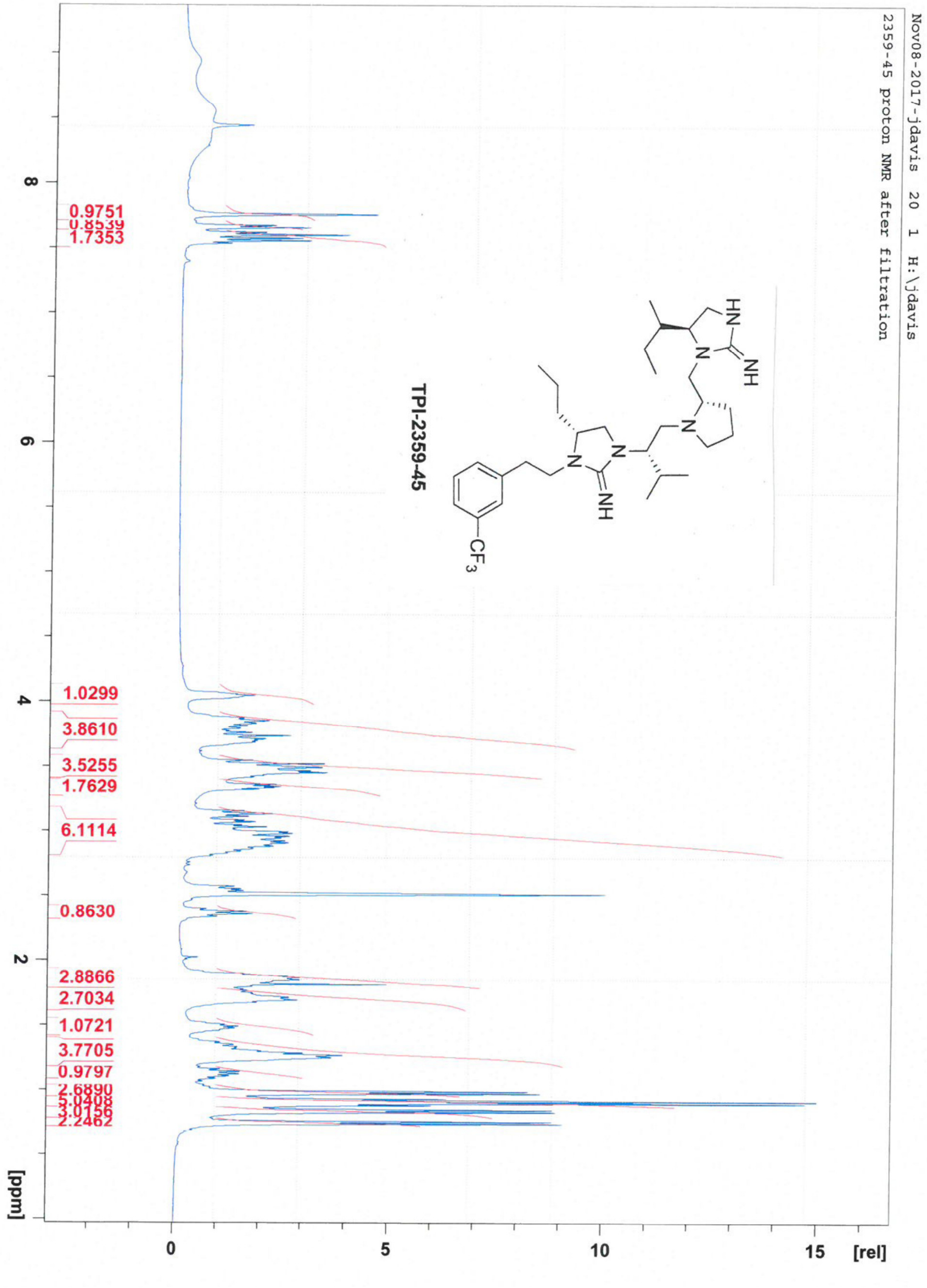

Nov08-2017-jdavis 20 1 H:\jdavis
2359-45 proton NMR after filtration
TPI-2359-45
HN
NH
N
CF3
0.9751
0.8539
1.7353
1.0299
3.8610
3.5255
1.7629
6.1114
0.8630
2.8866
2.7034
1.0721
3.7705
0.9797
2.6890
5.0408
3.0156
2.2462
8
6
4
2
[ppm]
0
5
10
15
[rel]

2539-45 pure C13

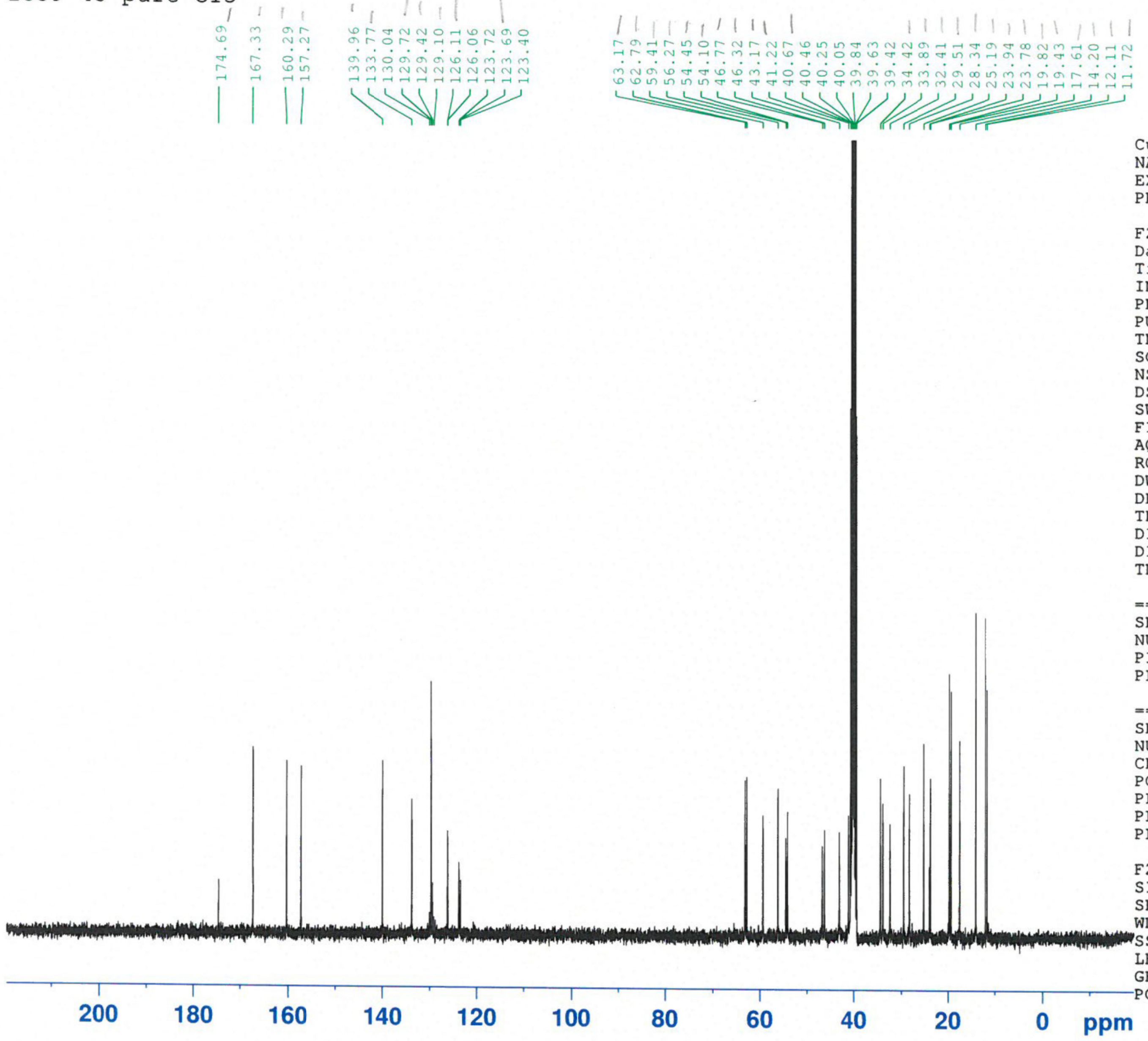

Current Data Parameters	
NAME	Oct23-2017-jdavis
EXPNO	61
PROCNO	1
F2 - Acquisition Parameters	
Date_	20171024
Time	2.16
INSTRUM	spect
PROBHD	5 mm PABBO BB/
PULPROG	zgpg30
TD	65536
SOLVENT	DMSO
NS	2048
DS	4
SWH	24038.461 Hz
FIDRES	0.366798 Hz
AQ	1.3631488 sec
RG	208.24
DW	20.800 usec
DE	6.50 usec
TE	299.8 K
D1	2.00000000 sec
D11	0.03000000 sec
TD0	1
======== CHANNEL f1 ========	
SFO1	100.6278588 MHz
NUC1	13C
P1	10.00 usec
PLW1	64.00000000 W
======== CHANNEL f2 ========	
SFO2	400.1516006 MHz
NUC2	1H
CPDPRG[2	waltz16
PCPD2	90.00 usec
PLW2	29.00000000 W
PLW12	0.35802001 W
PLW13	0.28999999 W
F2 - Processing parameters	
SI	32768
SF	100.6177980 MHz
WDW	EM
SSB	0
LB	1.00 Hz
GB	0
PC	1.40

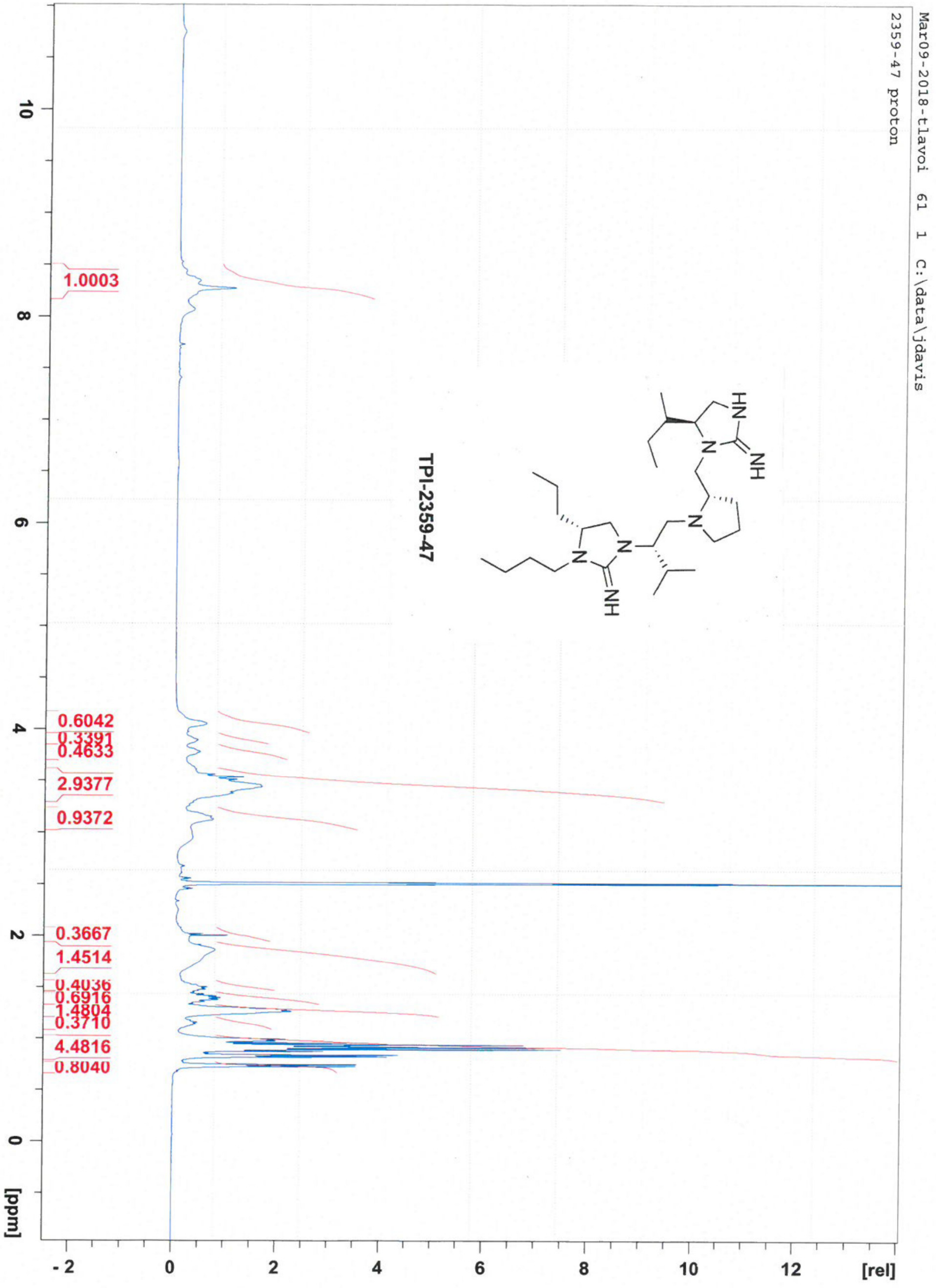
Mar09-2018-tlavoi 61 1 C:\data\jdavis
2359-47 proton
TPI-2359-47
NH
HN
N
N
N
N
NH
1.0003
0.6042
0.3391
0.4633
2.9377
0.9372
0.3667
1.4514
0.4036
0.6916
1.4804
0.3710
4.4816
0.8040
10
8
6
4
2
0
[ppm]
- 2
0
2
4
6
8
10
12
[rel]

2539-47 pure C13

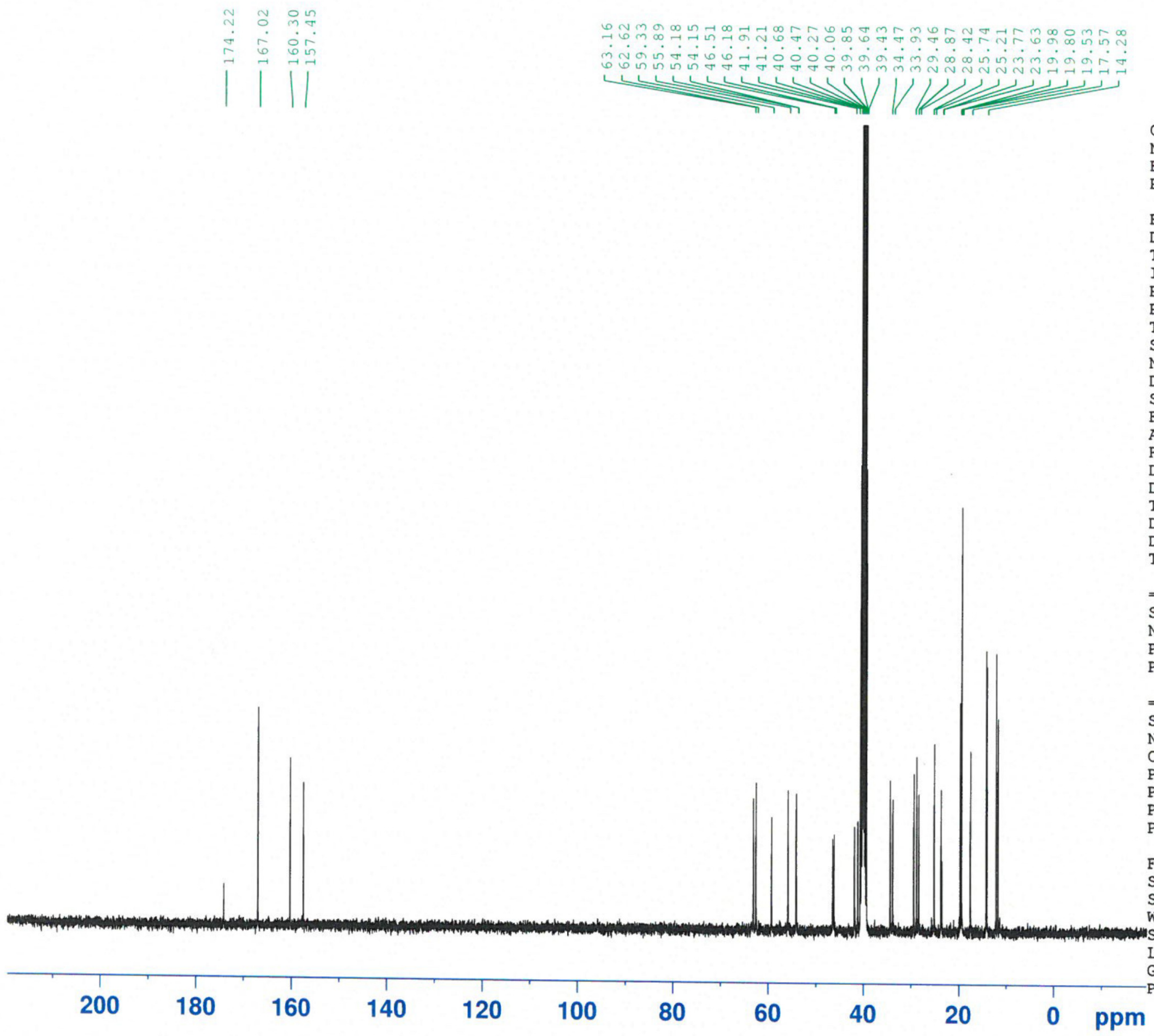


```
Current Data Parameters
NAME        Oct23-2017-jdavis
EXPNO                      71
PROCNO                      1

F2 - Acquisition Parameters
Date_                20171024
Time                     4.41
INSTRUM                 spect
PROBHD      5 mm PABBO BB/
PULPROG               zgpg30
TD                      65536
SOLVENT                  DMSO
NS                       2048
DS                          4
SWH                 24038.461 Hz
FIDRES               0.366798 Hz
AQ                  1.3631488 sec
RG                     208.24
DW                     20.800 usec
DE                       6.50 usec
TE                      299.7 K
D1                 2.00000000 sec
D11                0.03000000 sec
TD0                         1

======== CHANNEL f1 ========
SFO1             100.6278588 MHz
NUC1                      13C
P1                      10.00 usec
PLW1              64.00000000 W

======== CHANNEL f2 ========
SFO2             400.1516006 MHz
NUC2                       1H
CPDPRG[2              waltz16
PCPD2                   90.00 usec
PLW2              29.00000000 W
PLW12              0.35802001 W
PLW13              0.28999999 W

F2 - Processing parameters
SI                      32768
SF               100.6177980 MHz
WDW                        EM
SSB          0
LB                       1.00 Hz
GB           0
PC                       1.40
```

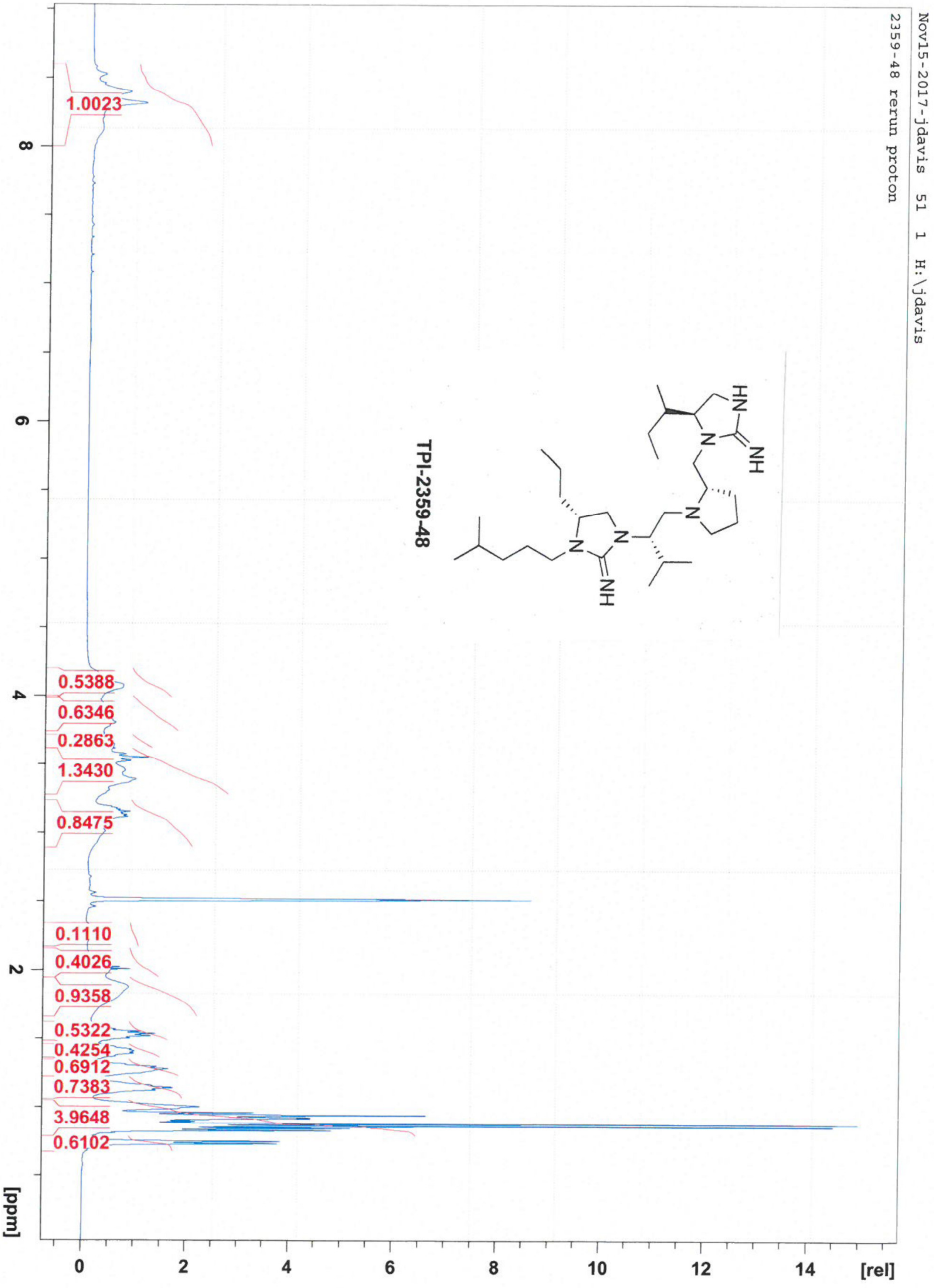

Nov15-2017-jdavis 51 1 H:\jdavis
2359-48 rerun proton
TPI-2359-48
NH
HN
N
NH
1.0023
0.5388
0.6346
0.2863
1.3430
0.8475
0.1110
0.4026
0.9358
0.5322
0.4254
0.6912
0.7383
3.9648
0.6102
8
6
4
2
[ppm]
0
2
4
6
8
10
12
14
[rel]

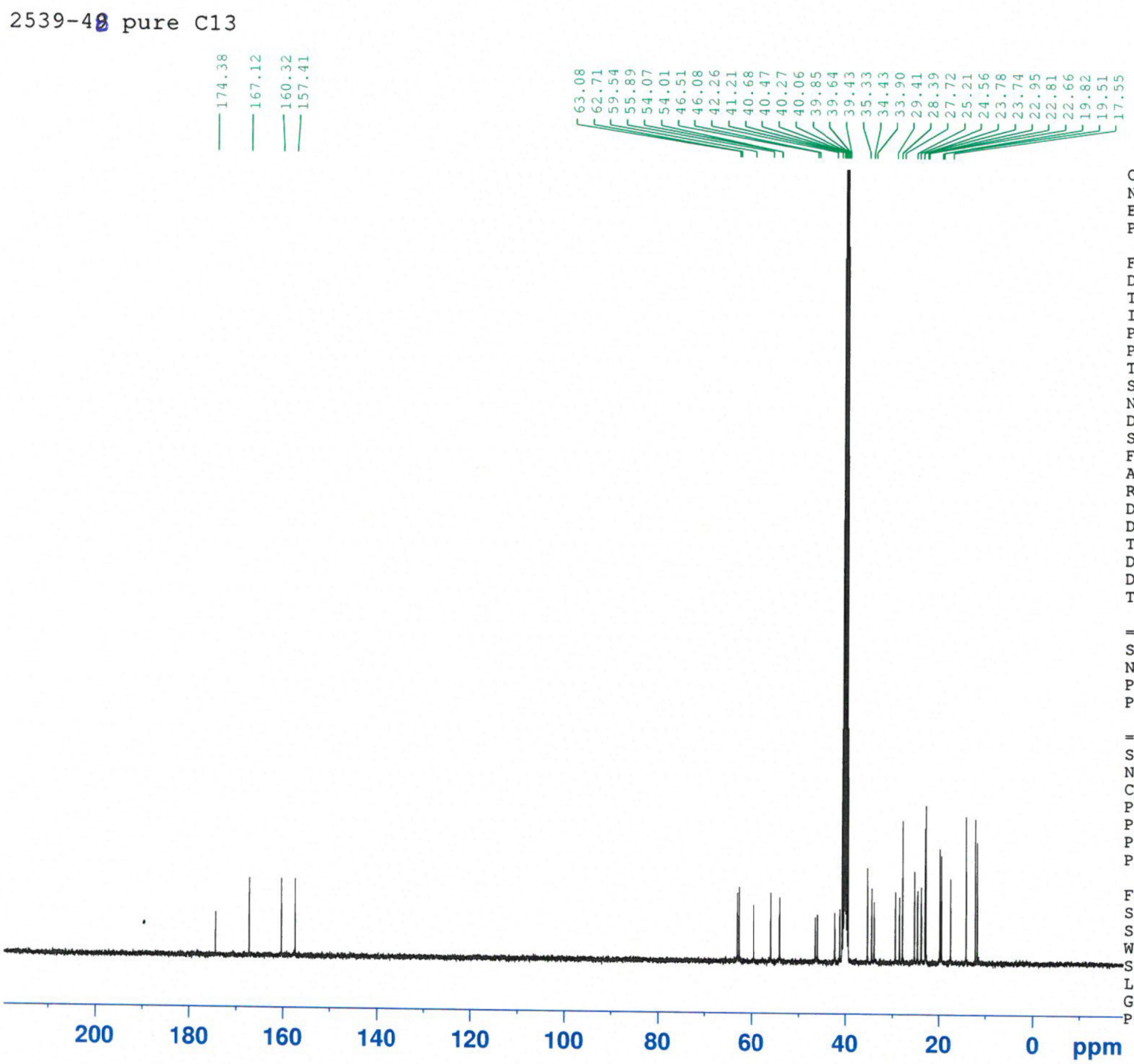


```
Current Data Parameters
NAME        Oct23-2017-jdavis
EXPNO                     81
PROCNO                     1

F2 - Acquisition Parameters
Date_               20171024
Time                    7.06
INSTRUM                spect
PROBHD      5 mm PABBO BB/
PULPROG              zgpg30
TD                    65536
SOLVENT                DMSO
NS                     2048
DS                        4
SWH               24038.461 Hz
FIDRES             0.366798 Hz
AQ                1.3631488 sec
RG                   208.24
DW                   20.800 usec
DE                     6.50 usec
TE                    299.7 K
D1               2.00000000 sec
D11              0.03000000 sec
TD0                       1

======== CHANNEL f1 ========
SFO1            100.6278588 MHz
NUC1                    13C
P1                    10.00 usec
PLW1            64.00000000 W

======== CHANNEL f2 ========
SFO2            400.1516006 MHz
NUC2                     1H
CPDPRG[2            waltz16
PCPD2                 90.00 usec
PLW2            29.00000000 W
PLW12            0.35802001 W
PLW13            0.28999999 W

F2 - Processing parameters
SI                    32768
SF              100.6177980 MHz
WDW                      EM
SSB          0
LB                     1.00 Hz
GB           0
PC                     1.40
```

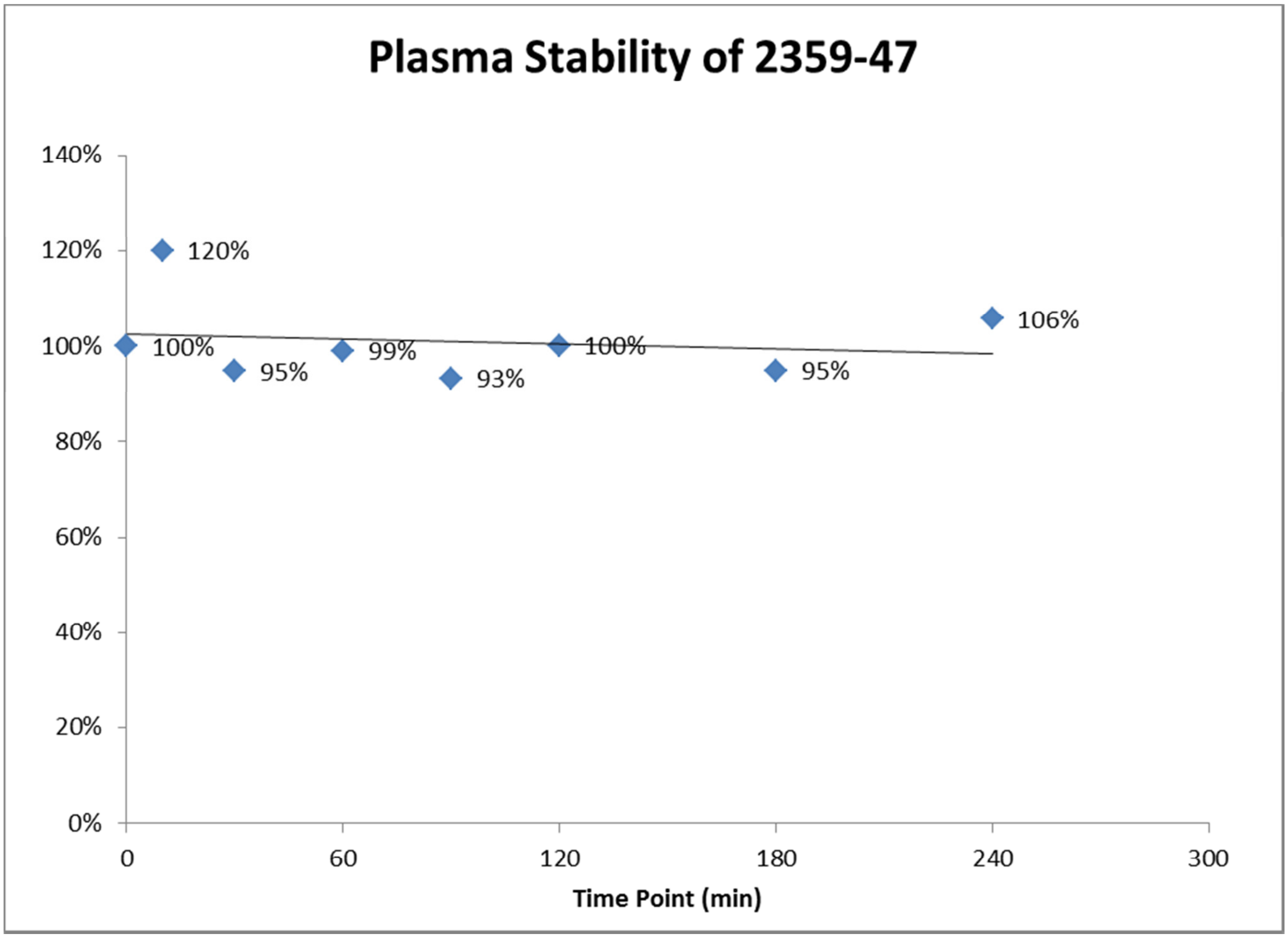

The percentage of analyte detected compared to time point zero.
